# Dihydroartemisinin inhibits mutant KRAS to potentiate regorafenib plus anti-PD-1 in *KRAS*-mutant colorectal cancer liver metastases

**DOI:** 10.1038/s41419-026-09042-z

**Published:** 2026-07-06

**Authors:** Ning Huang, Liming Wang, Hongming Deng, Chenyang Nan, Zhenbang Ye, Changchun Zhou, Jiajun Zhang, Ruoyu Zhang, Ke Xu, Jianxiong Wu, Rui Liu, Mei Liu

**Affiliations:** 1 Department of Hepatobiliary Surgery, National Cancer Center/National Clinical Research Center for Cancer/Cancer Hospital, Chinese Academy of Medical Sciences and Peking Union Medical College, Beijing, China; 2 Laboratory of Cell and Molecular Biology & State Key Laboratory of Molecular Oncology, National Cancer Center/National Clinical Research Center for Cancer/Cancer Hospital, Chinese Academy of Medical Sciences and Peking Union Medical College, Beijing, China; 3 Department of Pathology, National Cancer Center/National Clinical Research Center for Cancer/Cancer Hospital, Chinese Academy of Medical Sciences and Peking Union Medical College, Beijing, China; 4 Shandong Cancer Hospital and Institute, Shandong First Medical University and Shandong Academy of Medical Sciences, Shandong Provincial Key Laboratory of Precision Oncology, Jinan, China; 5 Tianjin Medical University Cancer Institute and Hospital, National Clinical Research Center for Cancer, Tianjin’s Clinical Research Center for Cancer, Key Laboratory of Cancer Prevention and Therapy, Tianjin Key Laboratory of Digestive Cancer, Tianjin, China

**Keywords:** Cancer immunotherapy, Cancer

## Abstract

Colorectal cancer (CRC) is one of the most prevalent malignancies worldwide, and liver metastases stands as a leading contributor to its high mortality rate in advanced stages. Regorafenib plus anti-PD-1 is a new therapeutic option for patients with colorectal cancer liver metastases (CRCLM). However, a considerable number of patients have not benefited from it. In this study, *KRAS* mutation was identified associated with resistance to regorafenib plus anti-PD-1 in CRCLM. Dihydroartemisinin (DHA), a clinically approved anti-malaria agent, was verified to selectively downregulate KRAS^G12D^ mutant with no discernible influences on wild-type KRAS, which is consistent with the higher sensitivity of *KRAS*-mutant CRC cells and organoids to DHA treatment. In preclinical *KRAS*^G12D^ CRCLM models, DHA substantially potentiated the therapeutic efficacy of regorafenib plus anti-PD-1 by remodeling the tumor immune microenvironment, including enhancing the cytotoxicity of CD8^+^ effector T cells and promoting pro-inflammatory macrophage polarization. Mechanically, DHA could restore interferon response that was impaired by oncogenic *KRAS* mutations, and inhibit ERBB signaling activation induced by regorafenib. Collectively, these findings support DHA as a potential adjunct to regorafenib plus anti-PD-1 for *KRAS*^G12D^ CRCLM and suggest a therapeutic strategy with translational potential for *KRAS*-driven malignancies.

**Figure:**
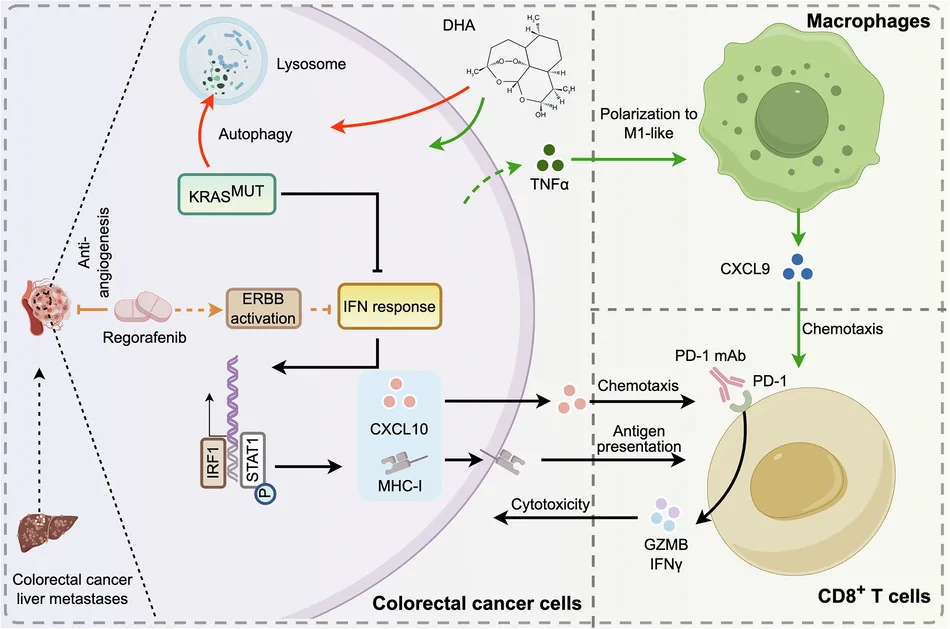
Black arrows: Mutant KRAS can impair IFN response to inhibit CD8^+^ T cells anti-tumor immune response, thus attenuating the efficacy of PD-1 mAb therapy. Red arrows: DHA can inhibit mutant KRAS expression by promoting autophagy-lysosome pathway. Orange arrows: Regorafenib plays a role of anti-angiogenesis, but also probably induces ERBB signaling activation, which can inhibit IFN response via upregulating p-Erk1/2 and p-Akt, thus causing resistance to regorafenib treatment. Green arrows: The treatment of DHA can drive macrophages polarization to pro-inflammation phenotype (M1-like), probably via the enhancement of TNFα expression. This Figure was produced by Figdraw.

Black arrows: Mutant KRAS can impair IFN response to inhibit CD8^+^ T cells anti-tumor immune response, thus attenuating the efficacy of PD-1 mAb therapy. Red arrows: DHA can inhibit mutant KRAS expression by promoting autophagy-lysosome pathway. Orange arrows: Regorafenib plays a role of anti-angiogenesis, but also probably induces ERBB signaling activation, which can inhibit IFN response via upregulating p-Erk1/2 and p-Akt, thus causing resistance to regorafenib treatment. Green arrows: The treatment of DHA can drive macrophages polarization to pro-inflammation phenotype (M1-like), probably via the enhancement of TNFα expression. This Figure was produced by Figdraw.

## Introduction

Colorectal cancer (CRC) metastases is the most leading lethal cause in patients, with the liver representing the most frequent site of secondary involvement [1, 2]. Due to the limitations of surgical intervention, patients with colorectal cancer liver metastases (CRCLM) are predominantly treated with chemotherapy, targeted therapy and immunotherapy. However, CRCLM is characterized by a high degree of genetic heterogeneity [3] and a highly suppressive tumor immune microenvironment (TIME) [4–6], for which a single-agent therapy regimen is usually of little benefit for patients. Recently, numerous clinical trials on targeted and immunotherapy combinations have been carried out worldwide. Previous studies have reported that microsatellite-stable tumors, which are conventionally considered insensitive to immune checkpoint blockade (ICB) therapy, can also be effectively inhibited by targeted therapy plus immunotherapy, for example, the use of regorafenib in conjunction with nivolumab [7, 8], whereas another trial, IMblaze370, reported that the efficacy of a combination of two drugs was not better than a single drug [9].

Regorafenib is a multi-targeted tyrosine kinase inhibitor that targets proteins closely associated with cell proliferation, including vascular endothelial growth factor receptor (VEGFR), platelet-derived growth factor receptor (PDGFR), fibroblast growth factor receptor (FGFR), and BRAF [10, 11]. Therefore, regorafenib has dual effects, including normalizing blood vessels within tumors thus promoting drug delivery and immune cells infiltration, and directly inhibiting cancer cells proliferation by targeting kinase like BRAF. In real-world clinical practices, we tried to employ the combination of regorafenib and PD-1 monoclonal antibody (mAb) to treat CRCLM patients, but disease remission was achieved in only a proportion of patients. To elucidate the underlying mechanisms of therapeutic resistance, we analyzed their clinical and pathological characteristics, and found that most non-responders obtained *KRAS* mutations. Approximately 50% of CRC patients feature *KRAS* mutations, with *KRAS*^G12D^ as the most prevalent mutant type, followed by *KRAS*^G12V^ and *KRAS*^G13D^ [12, 13]. Mutant KRAS induces a sustained activation of its downstream signaling, such as p-Erk1/2 and p-Akt, which could result in resistance to multiple drugs, through mediating bypass signaling pathways, suppressing anti-tumor immune responses, inducing secondary mutations, etc [14–16].

KRAS was initially regarded as an “undruggable” target because of its smooth protein surface. However, since 2013, when the first small molecule was developed to target KRAS^G12C^ and to induce apoptosis [17], the exploration of strategies targeting mutant KRAS has been one of the major areas of cancer research. Currently available clinically approved inhibitors are restricted to KRAS^G12C^ and are primarily indicated for lung cancer [18], but there are no approved drugs for CRC with *KRAS*^G12D^, *KRAS*^G12V^ and *KRAS*^G13D^ mutations. On the other hand, resistance to sotorasib (targeting KRAS^G12C^) and MRTX1133 (targeting KRAS^G12D^) can emerge during the course of CRC treatment largely due to compensatory activation of the EGFR signaling pathway [19, 20], which is also a possible reason for the resistance to regorafenib in *KRAS*-mutant CRC. Therefore, novel options of mutant KRAS-targeted drugs are urgently needed.

In this study, we revealed the mechanism by which *KRAS* mutation mediated the poor sensitivity to regorafenib plus anti-PD-1 in CRCLM. More importantly, we identified that dihydroartemisinin (DHA), a natural small molecule used for anti-malaria, could downregulate KRAS^G12D^ expression in CRC and remodel the TIME, thereby potentiating regorafenib plus anti-PD-1 in CRCLM. Furthermore, we thoroughly elucidated the corresponding mechanisms involved. Since DHA is a clinically approved drug, these findings lay a solid foundation and provide a promising choice for CRCLM treatment.

## Methods and materials

### Clinical samples collection

Sixteen CRCLM cases were collected to investigate the molecular pathological characteristics **(**Supplementary Table 1**)**. Tumor tissues from six patients, including three responders (*KRAS* wild type, *KRAS*^WT^) and three non-responders (*KRAS* mutation, *KRAS*^MUT^) to regorafenib plus anti-PD-1, were utilized for bulk transcriptome sequencing (RNA-seq). Paraffin-embedded CRCLM tissues from eighteen patients were used for immunohistochemistry (IHC) staining or multiple immunofluorescence (mIF) staining.

### Bulk RNA-seq and data analyses

Total RNA of tumor tissues from human was extracted with the RecoverAll™ Total Nucleic Acid Isolation Kit for FFPE (AM1975, ThermoFisher), and that from mice was extracted with TRIzol. The RNA sequencing was conducted by BGI (Wuhan, China). Functional enrichment analyses were performed by Gene Ontology (GO), Kyoto Encyclopedia of Genes and Genomes (KEGG) and Gene Set Enrichment Analysis (GSEA). Immune infiltration was assessed using CIBERSORT algorithm.

### Cell lines and cell cultures

LS180 (CL-187, RRID:CVCL_0397), MC38 (RRID:CVCL_B288) and Caco-2 (RRID:CVCL_0025) cells were purchased from the Cell Resource Center, Institute of Basic Medical Sciences, Chinese Academy of Medical Sciences. HCT116 (CCL-247, RRID:CVCL_0291), SW480 (CCL-228, RRID:CVCL_0546) and CT26 (CRL-2638, RRID:CVCL_7254) cells were purchased from the American Type Culture Collection (Manassas, VA, USA). HT29 cells (HTB-38, RRID:CVCL_A8EZ) were kindly provided by Prof. Hongying Wang, and were purchased from the American Type Culture Collection (Manassas, VA, USA). According to the Cancer Cell Line Encyclopedia, LS180 cells harbor *KRAS*^G12D^ mutation, SW480 cells harbor *KRAS*^G12V^ mutation, HCT116 cells harbor *KRAS*^G13D^ mutation, and Caco-2 and HT29 cells are *KRAS*^WT^ cells. Previous studies have reported that CT26 cells have *Kras*^G12D^ mutations, while MC38 cells are *Kras*^WT^ cells [21, 22]. LS180, SW480, HCT116, HT29, CT26 and MC38 cell lines were cultured in RPMI 1640 containing 10% fetal bovine serum (FBS) and 1% penicillin/streptomycin. Caco-2 cells were cultured in DMEM with 10% FBS and 1% penicillin/streptomycin. All the cells were cultured in a humidified environment at 37°C and with 5% CO_2_. The cells were passaged every two or three days, and were tested for Mycoplasma contamination with the MycoGuard™ Mycoplasma PCR Detection Kit (MP004, GeneCopoeia, USA) every 3 months.

### Plasmids, stable cells construction and transient transfection

MC38 cells were used for constructing mouse Kras^G12D^ over-expression cells, and the pLV3-CMV-*Kras*^G12D^-Puro plasmid was purchased from MiaoLingPlasmid, China. Caco-2 and HT29 cells were used for constructing human KRAS^G12D^ over-expression cells, and the pLVX-EF1α-*KRAS*^G12D^-IRES-Puro plasmid was obtained from Beijing Likeli Biotechnology. The lentivirus packaging plasmids pMD2.G (#12259) and psPAX2 (#12260) were purchased from Addgene (Massachusetts, USA). Lentiviruses were produced by transfecting HEK293T cells, using Neofect DNA transfection reagent (Neofect biotech, Beijing), and with the proportion of 7:2:5 of lentiviral constructs, pMD2.G and psPAX2. The cells were infected with lentivirus, which was promoted by 10 μg/mL Polybrene (TR-1003, Sigma-Aldrich). The infected cells were selected with puromycin (6 μg/mL for MC38 cells, 2 μg/mL for Caco-2 and HT29 cells). Transient transfection was performed with Lipofectamine 3000 kit (L3000015, ThermoFisher) according to the manufacturer’s instructions. Briefly, cells were seeded in 6-well plates, and plasmids pLV3-CMV-*KRAS*^G12D^-Puro, pLV3-CMV-*KRAS*^G12D/S145A^-Puro, pLV3-CMV-*KRAS*^G12D/S145G^-Puro (MiaoLingPlasmid, China) were transfected into cells after cell adhesion.

### Mice models and therapy

Female C57BL/6 J and BALB/c mice (4 weeks old) were purchased from HFK BIOSCIENCE (Beijing, China). 1×10^6^ MC38 cells or CT26 cells suspended in 100 μL of phosphate-buffered saline (PBS) were injected via mice spleen to construct CRCLM models.

Female BALB/c nude mice (4 weeks old) were purchased from Vital River (Beijing, China). 2×10^6^ HCT116 cells, suspended in 100 μL of PBS with 50% Matrix gel (356234, Corning, USA), were inoculated subcutaneously under the left axilla. The treatment commenced when the tumors grew up to approximately 50mm^3^ (tumor size = 1/2×length×width^2^).

Regorafenib (HY-10331, MedChemExpress) and DHA (HY-N0176, MedChemExpress) were given intragastrically with corn oil, and PD-1 mAb (A2122, Selleck) was administered via intraperitoneal injection. Mice bearing subcutaneous tumors were treated daily, while those with orthotopic tumors received treatment once every three days.

### Western blot

Cells were lysed in RIPA buffer (R0010, Solarbio, Beijing, China). Western blot assay was performed as described before [23]. The primary antibodies used were listed in Supplementary Table 2. After being incubated with secondary antibodies at room temperature for one hour and washed with TBS-T, the membranes were exposed in the ImageQuant 800 system to detect the protein levels.

### Multiple immunofluorescence (mIF) staining

The mIF staining was performed using a seven-color mIF Kit (AFIHC037, Hunan Aifang Biological Technology, China), which is based on the tyramide signal amplification (TSA) technology. The experiments were conducted according to the manufacturer’s instructions. The primary antibodies used were listed in Supplementary Table 2. The captured images were analyzed with ImageJ, and the mean fluorescence intensity (MFI) or the proportion of positive cells was estimated.

### Drug virtual screening and molecular dynamics simulation

The KRAS^G12D^ protein structure (7EYX) was downloaded from the Protein Data Bank (https://www.rcsb.org/). The library of natural active compounds from traditional Chinese medicine were obtained from MedChemExpress. Virtual screening was performed with the Glide module in Schrödinger Maestro 12.8 according to the tutorials, and a more negative score indicates a stronger predicted binding.

The molecular dynamics simulation was performed with Gromacs2022. For system setup, DHA was parameterized with the GAFF force field, the KRAS^G12D^ protein with the AMBER14SB force field, and the TIP3P water model was used as solvent. The DHA-KRAS^G12D^ complex was placed in a box under periodic boundary conditions, and counterions were added to neutralize the system charge. Energy minimization was conducted via the steepest descent method to eliminate steric clashes. Sequentially, 100 ps NVT and 100 ps NPT equilibrations were performed at 298 K, with temperature controlled by the V-rescale thermostat and pressure maintained at 1 bar using the Berendsen barostat during NPT equilibration. A 100 ns production simulation was then carried out, with pressure controlled at 1 bar via the Parrinello–Rahman barostat. All H-containing bonds were constrained using the LINCS algorithm, with a 2 fs integration time step. The Verlet cutoff scheme was used for neighbor list updating, electrostatic interactions were calculated by the PME method, and a 10 Å cutoff was applied for both electrostatic and van der Waals interactions. Conformations were saved every 10 ps. Trajectory analysis was performed using VMD and PyMOL software.

### Surface Plasmon Resonance (SPR) assay

KRAS^G12D^ protein (R06-32BH, Sino Biological, China) was immobilized onto a CM5 sensor chip (Cytiva) using amine coupling chemistry. Solvent correction solutions were prepared and a correction curve was established. A concentration gradient of DHA (HY-N0176, MedChemExpress) was prepared. DHA solutions from low to high concentrations were injected sequentially over the chip surface to analyze the protein-drug interaction, with an association phase of 60 s, a dissociation phase of 180 s, and chip regeneration between each concentration. Data were processed using Biacore Insight software to obtain relevant kinetic parameters. K_D_ represents the dissociation constant, reflecting the binding affinity between the ligand and receptor; a lower K_D_ value indicates stronger binding affinity. Ka is the association rate constant, describing the speed of complex formation; a higher Ka value indicates faster binding. Kd is the dissociation rate constant, describing the stability of the complex; a higher Kd value indicates faster dissociation.

### Half maximal inhibitory concentration (IC_50_) estimation

Cells were seeded into 96-well plates at a density of 1×10^4^ cells per well. After the cells adhered to the plates, a concentration gradient of drugs (0 μM, 0.39 μM, 0.78 μM, 1.56 μM, 3.125 μM, 6.25 μM, 12.5 μM, 25 μM, 50 μM, and 100 μM regorafenib; 0 μM, 0.01 μM, 0.1 μM, 1 μM, 10 μM, 20 μM, 40 μM, 60 μM, 80 μM, and 100 μM DHA) were added. 48 h later, the drugs were removed and 100 μL of cell culture medium containing 10% CCK-8 solution (CK04, DOJINDO, Japan) was added for a 1.5 h incubation. The absorbance was measured at 450 nm with microplate reader (Bio-Rad). The IC_50_ values were calculated with GraphPad Prism 8.0.

### Synergistic analyses of DHA and regorafenib

According to the estimated IC_50_ values, a series of concentrations of DHA and regorafenib were set for analysing their synergistic effects. The analyses were conducted with SynergyFinder, and measured by the HSA synergy score (with a threshold of 10), with a higher HSA synergy score indicating a stronger synergistic effect.

### Organoids construction and culture

The CRC organoids were constructed and cultured in accordance with the method described before [24]. Tumor tissues were mechanically dissected and digested with digestion medium and DNase. Samples were filtered through a 70 μm cells strainer, and centrifuged at 270×g for 5 min at 4°C. Cells were resuspended with DMEM/F12, mixed with Matrigel and seeded into cell culture plate at an appropriate volume. After gelation at 37°C and adding CRC organoid culture medium, the plate was placed at 37°C, 5% CO_2_ incubator.

For organoids passage, the medium was removed and 4°C DMEM/F12 was added to dissociate the Matrigel. The pellet was pipetted several times and TrypLE Express was used for releasing and dissociating organoids into single cells. After being centrifuged at 270×g for 5 min at 4°C, the cells were resuspended in DMEM/F12 and finally mixed with Matrigel and seeded into the plate.

### Colony formation assay

LS180, SW480 and HCT116 cells were seeded into 6-well plates at densities of 1000 cells/well, 500 cells/well and 500 cells/well, respectively, and treated with conditional media containing different drugs (regorafenib, DHA or both regorafenib and DHA). After an incubation for 10–15 day, the cells were fixed with 4% paraformaldehyde (PFA) and stained with crystal violet. ImageJ was used for colonies counting.

### Apoptosis assay by flow cytometry

The Annexin V-APC/PI kit (P-CA-207, Procell, Wuhan, China) was used for apoptosis assay. The experiment was conducted according to the manufacturer’s instructions. FlowJo V10 was used for flow cytometry analysis. The proportions of all APC^+^ cells (including early apoptosis, late apoptosis and necrosis) were used for statistical analysis.

### Hematoxylin and eosin (H&E) staining

Sections were deparaffinized and rehydrated. Hematoxylin staining was performed, followed by differentiation in 1% acid alcohol. The sections were then blued in ammonia water and subjected to eosin staining. The sections were subsequently dehydrated, cleared in xylene, and mounted with neutral balsam.

### Immunohistochemistry (IHC) staining

Paraffin-embedded tissue slides were deparaffinized and hydrated, and then performed heat-induced antigen retrieval. Primary antibodies **(**Supplementary Table 2**)** were diluted to an appropriate concentration for incubation at 4°C overnight. After incubation by the secondary antibodies for 1 h at room temperature, DAB was used for staining. The H-scores, counted as [(weak intensity cells%)×1 + (moderate intensity cells%)×2 + (strong intensity cells%)×3]×100, were estimated by QuPath.

### TUNEL assay

The TUNEL assay kit (E-CK-A321, Elabscience, Wuhan, China) was applied to detect apoptosis in situ, following the instructions of manufacturer. The images were analyzed by ImageJ.

### Single-cell transcriptome sequencing (scRNA-seq) and analyses

Single-cell suspension preparation, library preparation and scRNA-seq were conducted by CapitalBio techonology (Beijing, China) based on 10× Genomics platform. Transcript mapping, and assignment to individual cells were performed with the STAR algorithm of Cell Ranger. The expression matrix was analyzed with Seurat V5, including differentially expressed genes (DEGs), cell annotation, GSEA and gene set variation analysis (GSVA).

### CD8^+^ T cells isolation, expansion and co-culture with cancer cells

Human peripheral blood lymphocytes (PBLCs) were isolated with separation medium (LTS 1077, tbdscience, Tianjin, China). CD8^+^ T cells were sorted from PBLCs with the CD8^+^ T Cell Isolation Kit (130-096-495, Miltenyi Biotech, Germany). CD8^+^ T cells were cultured and expanded in ImmunoCult™-XF T Cell Expansion Medium (10981, STEMCELL Technologies, Canada) with ImmunoCult™ Human CD3/CD28 T cell activator (10991, STEMCELL Technologies, Canada) and human interleukin (IL) -2 (100 IU/mL, Beijing Four Rings Bio-Pharmaceutical). The purity of isolated CD8^+^ T cells was verified by flow cytometry.

CD8^+^ T cells (effector) and cancer cells (target) were co-cultured at an effector: target ratio of 5:1, and with different conditional media with SCH772984 (p-Erk1/2 inhibitor, S7101, Selleck), MK-2206 2HCl (p-Akt inhibitor, S1078, Selleck), DHA or PD-1 mAb (pembrolizumab, MSD). Cellular immunofluorescence staining was performed to detect CD8^+^ T cells chemotaxis to cancer cells. The supernatant of the co-culture system was collected for enzyme-linked immunosorbent assay (ELISA) to detect CXCL10 (RK00054, ABclonal), GZMB (RK00089, ABclonal), and interferon γ (IFNγ) (RK00015, ABclonal), and for lactate dehydrogenase (LDH) determination (LDH Cytotoxicity Assay Kit, C0017, Beyotime) to assess cytotoxicity. Both ELISA and LDH determination were conducted following the manufacturers’ instructions.

### Cellular immunofluorescence (cIF) staining

Cells were fixed with 4% PFA for 20 min, and permeabilized with 0.5% Triton-X 100 for 15 min. The cells were subsequently incubated with primary antibodies (listed in Supplementary Table 2) at 4°C overnight and incubated with secondary antibodies at room temperature for 1 h. DAPI (G1012, Servicebio, Wuhan, China) was used for nuclei staining. The slides were covered with antifade mountant (ZLI-9556, ZSGB-BIO, Beijing, China). Images were analyzed with ImageJ.

### Statistical analyses

Data were shown in the form of mean ± SEM. The comparisons among different groups were performed with Student’s t-tests, One-Way ANOVA, Chi-Squared Test, or Fisher exact test. *P* < 0.05 was considered statistically significant in general, and in Chi-Squared Test among three groups, *p* < 0.017 was considered statistically significant with Bonferroni correction. The visualization was performed with R 4.4.1.

## Results

### *KRAS* mutation was associated with poor response to regorafenib plus anti-PD-1 in CRCLM

**Fig. 1:**
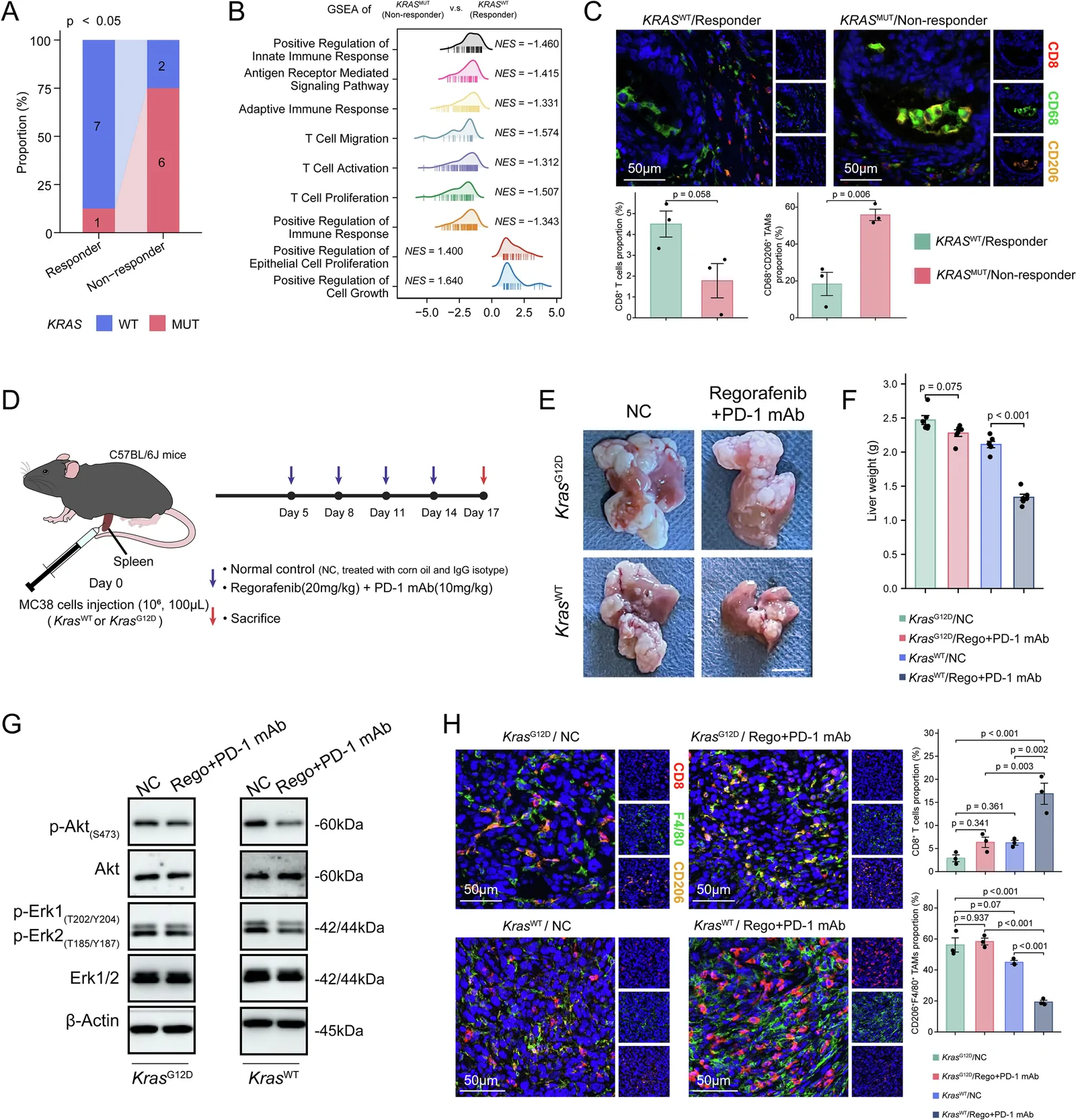
*KRAS* mutation contributes to the resistance to regorafenib and PD-1 mAb combination in CRCLM. **A** The *KRAS* status in CRCLM responders and non-responders to regorafenib plus anti-PD-1 therapy. **B** Ridge plot of GSEA of the RNA-seq conducted with clinical samples. The curves reflect genes distribution in each gene set. **C** The TIME profile detected by mIF in patients’ tumor tissues (*n* = 3 in each group). CD68 marks total macrophages. **D** The experimental workflow of C57BL/6 J mice CRCLM models bearing MC38 cells (*n* = 6 in each group). **E, F** Livers from CRCLM mice models and the statistical analysis of liver weight. **G** Western blot showing the downstream signaling pathways (p-Erk1/2 and p-Akt) of KRAS in mice models tumor tissues. **H** The TIME profile of mice models’ tumor tissues (*n* = 3 in each group). F4/80 marks total macrophages. GSEA Gene Set Enrichment Analysis, NES Normalized Enrichment Score, NC Normal Control, mAb monoclonal antibody, TIME tumor immune microenvironment.

In clinical practices, we observed that patients with CRCLM responded differently to regorafenib plus anti-PD-1. In order to find out the possible factors which could impact the treatment efficacy, we reviewed the molecular pathological records of sixteen clinical cases, with eight responders and eight non-responders to regorafenib plus anti-PD-1, and found that more cases were detected with mutant *KRAS* among non-responders **(**Fig. 1A**)**. Then, we selected well-stored specimens from three cases respectively in each group for bulk RNA-seq. The functional enrichment analyses showed that some biological processes, including anti-tumor immunity and cell growth, presented different activities between two groups **(**Fig. 1B**)**. Compared with *KRAS* wild-type tumors from responders, tumors from non-responders harboring *KRAS* mutation manifested lower immune responses and higher epithelial cell proliferation activity. TIME detection by mIF in situ also revealed more CD8^+^ T cells and less CD206^+^ tumor-associated macrophages (TAMs) infiltration in responders’ (*KRAS*^WT^) tumor tissues **(**Fig. 1C**)**.

With MC38 cells (*Kras*^WT^ and *Kras*^G12D^) **(**Supplementary Fig. 1A**)**, we constructed CRCLM mice models, which were then treated as the planned regimen **(**Fig. 1D**)**, and the body weight of mice was measured regularly **(**Supplementary Fig. 1B**)**. As was expected, after the intervention of regorafenib plus PD-1 mAb, *Kras*^WT^ tumor burdens decreased significantly, while no significant difference was observed in *Kras*^G12D^ tumor burdens **(**Fig. 1E, F**)**. The downstream signaling pathways by which KRAS regulates cell growth, such as p-Erk1/2 and p-Akt, were not significantly inhibited by therapy in *Kras*^G12D^ tumors, compared with *Kras*^WT^ tumors **(**Fig. 1G**)**. Additionally, in contrast to *Kras*^G12D^ tumors, regorafenib plus PD-1 mAb dramatically led to more CD8^+^ T cells and less CD206^+^ TAMs recruitment to *Kras*^WT^ tumors **(**Fig. 1H**)**.

Taken together, these findings revealed the important role of mutant *KRAS* in impacting the sensitivity of CRCLM to the combination of regorafenib and PD-1 mAb.

### DHA could be a candidate to downregulate mutant KRAS expression

**Fig. 2:**
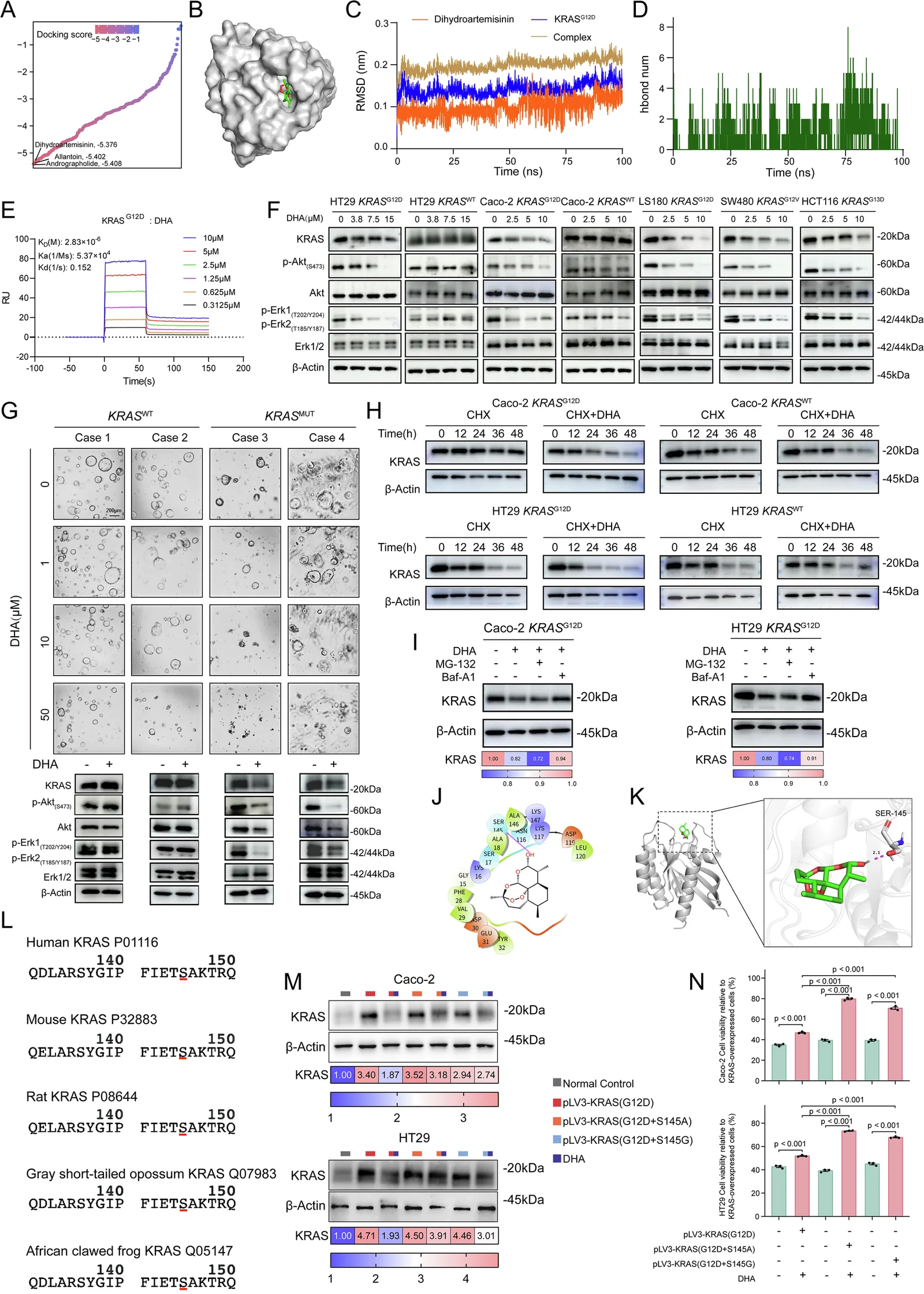
DHA inhibits mutant KRAS and the downstream signaling pathways. **A** Virtual screening of drugs targeting KRAS^G12D^. **B** DHA potential binding site on KRAS^G12D^. **C** RMSD plots of DHA, KRAS^G12D^, and the DHA-KRAS^G12D^ complex over 100 ns of molecular dynamics simulation, reflecting the structural stability of the system. **D** Time-dependent changes in the number of hydrogen bonds between DHA and KRAS^G12D^, indicating the stability of intermolecular interactions during the simulation. **E** Surface Plasmon Resonance assay of KRAS^G12D^ and DHA interaction. **F** Western blot validating the inhibition of KRAS^MUT^ or KRAS^WT^ and the downstream signaling pathways by concentration gradient of DHA. **G** Bright-field images of *KRAS*^WT^ (Case 1 & 2) and *KRAS*^MUT^ (Case 3 & 4) CRC organoids treated with 0–50 μM DHA. Western blots show levels of KRAS, p-Akt and p-Erk1/2 in organoids treated with DHA or not. **H** DHA accelerates the degradation of KRAS^G12D^ with CHX applied to block the protein synthesis. **I** The degradation of KRAS^G12D^ can be rescued by Baf-A1, an autophagy inhibitor. **J, K** Molecular docking reveals that DHA can interact with some amino acids in KRAS^G12D^, including forming hydrogen bond with Ser-145. **L** Part of KRAS amino acid sequence across different species from UniProt database. **M** The KRAS^G12D^ downregulation induced by DHA can be impaired with the presence of S145A or S145G mutation. **N** The presence of S145A or S145G mutation in KRAS can impair the effect of DHA on the inhibition of proliferation of cells. RMSD Root-Mean-Square Deviation, DHA dihydroartemisinin.

In order to seek new strategies of high translational value to improve the therapeutic efficacy, we introduced a compound library including 225 kinds of natural active small molecules from traditional Chinese medicine to find a possible one targeting KRAS^G12D^. In the result of virtual screening, DHA ranked after andrographolide and allantoin. **(**Fig. 2A, Supplementary Table 3**)**. Based on the research foundation and pharmacokinetic characteristics of the candidate compounds, DHA was selected for further validation (detailed elaboration is provided in the Discussion section), and it possibly binds to a certain site on KRAS^G12D^ protein **(**Fig. 2B**)**. In molecular dynamics simulation, the Root-Mean-Square Deviation (RMSD) of the DHA-KRAS^G12D^ protein complex rose slightly in the initial nanoseconds and then stabilized with narrow fluctuations **(**Fig. 2C**)**. Likewise, despite fluctuations in hydrogen bond count, the hydrogen bond network remained intact thereafter to sustain the intermolecular interaction **(**Fig. 2D**)**. We then conducted SPR assay to further validate the interaction between KRAS^G12D^ and DHA, and the result showed that they exhibited favorable binding affinity (K_D_ = 2.83×10^-6 ^M) **(**Fig. 2E**)**.

Caco-2 cells and HT29 cells are two *KRAS* wild-type cell lines, with which we constructed *KRAS*^G12D^ cells to investigate whether DHA could regulate the expression of *KRAS* **(**Supplementary Fig. 1C**)**. The IC_50_ values of several CRC cell lines showed that *KRAS*^WT^ cells were less sensitive to DHA than *KRAS*^MUT^ cells, particularly between *KRAS*^WT^ and *KRAS*^G12D^ Caco-2 and HT29 cells **(**Supplementary Fig. 1D**)**, which potentially indicated that the mutant KRAS could be a target of DHA. Cells treated with a concentration gradient of DHA, we found that KRAS^G12D^ was obviously downregulated, while the KRAS^WT^ was not, and the downstream pathways, such as p-Erk1/2 and p-Akt, showed similar tendencies **(**Fig. 2F**)**. Beyond expectations, DHA also efficiently inhibited the expression of *KRAS* in LS180 (*KRAS*^G12D^), SW480 (*KRAS*^G12V^) and HCT116 (*KRAS*^G13D^) cells **(**Fig. 2F**)**. With CRC organoids constructed with tissues from four patients, we evaluated the efficacy of DHA in inhibiting CRC, and observed that DHA exerted a more potent inhibitory effect on organoids harboring *KRAS* mutations, along with much more downregulation of KRAS expression and downstream p-Akt and p-Erk1/2 **(**Fig. 2G**)**.

Based on the molecular docking results and the decrease in the protein level of KRAS^G12D^, we speculated that DHA might induce the degradation of KRAS^G12D^. Using cycloheximide (CHX), a protein synthesis inhibitor, we found DHA accelerated the degradation of KRAS^G12D^, rather than KRAS^WT^ **(**Fig. 2H**)**, which could be rescued by Baf-A1 (an autophagy-lysosome inhibitor) instead of MG-132 (a proteasome and calpain inhibitor) **(**Fig. 2I**)**. Molecular docking revealed that DHA could have interactions with some amino acid residues **(**Fig. 2J**)**, including forming hydrogen bonds, and one of the most representative residues is serine (Ser)-145 **(**Fig. 2K**)**. Additionally, the Ser-145 residue is highly conserved across species **(**Fig. 2L**)**. In order to explore whether Ser-145 is essential for DHA-induced KRAS^G12D^ degradation, we ectopically expressed KRAS^G12D^ protein or double mutants KRAS with G12D/S145A (Ser-145 mutated to alanine) or G12D/S145G (Ser-145 mutated to Glycine). After treated with DHA for 48 h, the double mutants KRAS was less downregulated than the single mutant **(**Fig. 2M**)**, and the proliferation of cells harboring double mutants were less inhibited by DHA **(**Fig. 2N**)**. These results indicated that DHA can specifically suppress the expression of KRAS^G12D^ by inducing its degradation via autophagy-lysosome pathway and potentially dependent on Ser-145.

### DHA could synergize with regorafenib to inhibit colorectal cancer

**Fig. 3:**
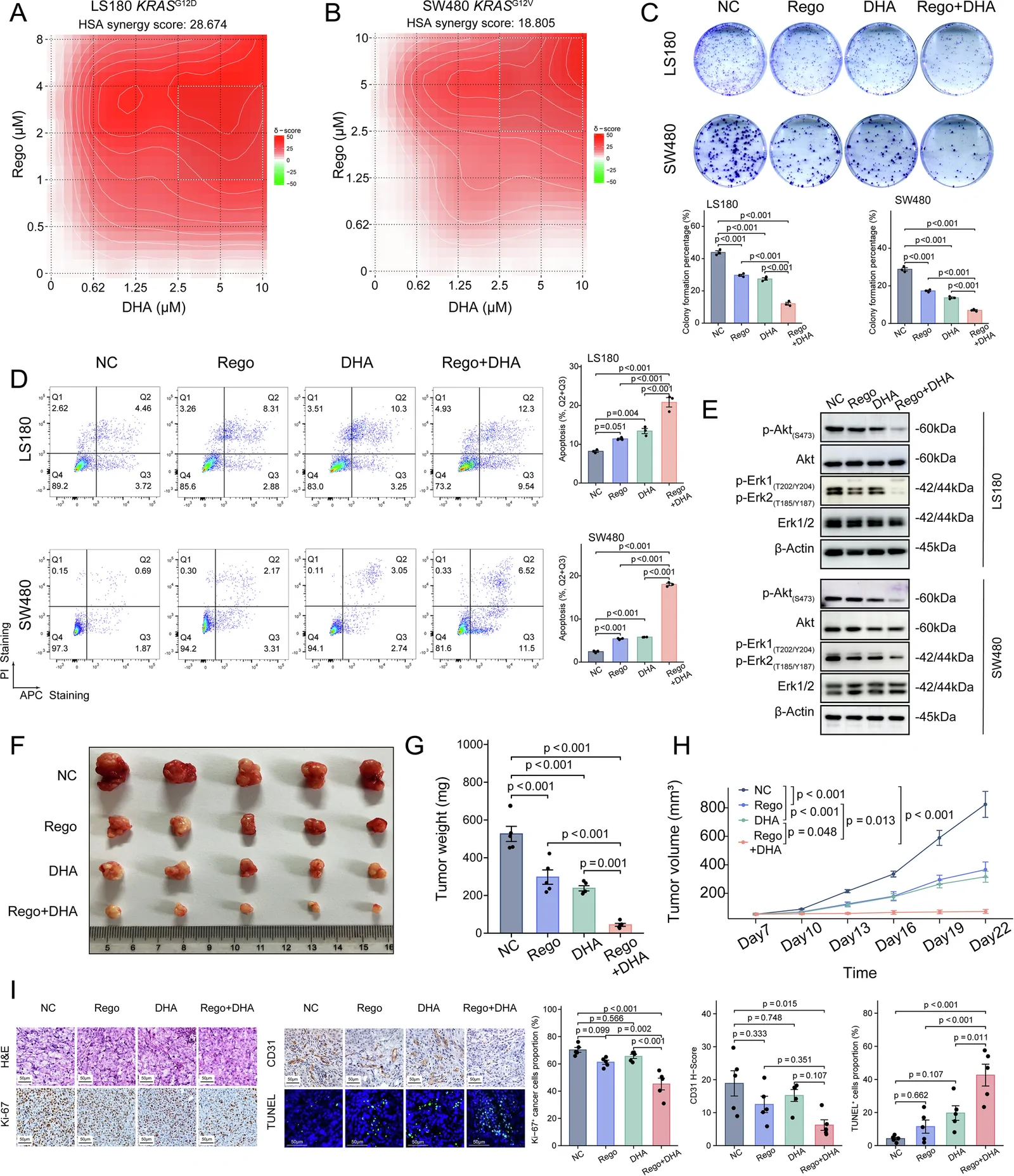
DHA synergizes with regorafenib to suppress CRC both in vitro and in vivo. **A, B** Synergistic analyses of regorafenib and DHA in LS180 and SW480 cells. **C** The synergistic anti-proliferation effect of regorafenib and DHA assessed by colony formation assay (*n* = 3 replicates). **D** The synergistic pro-apoptosis effect of regorafenib and DHA detected by flow cytometry with APC and PI staining (*n* = 3 replicates). **E** Western blot validating the synergistic inhibition of p-Erk1/2 and p-Akt by regorafenib and DHA. **F** The subcutaneous tumors in BALB/c nude mice models (*n* = 5 in each group). **G** The tumor weight upon sampling. **H** Changes in tumor size during the experimental process. **I** H&E, IHC and TUNEL staining of the tumor tissues to assess proliferation (Ki-67), angiogenesis (CD31) and apoptosis (TUNEL) (*n* = 5 in each group). NC Normal Control, Rego regorafenib, DHA dihydroartemisinin.

To investigate the influence of *KRAS* mutation on the effect of regorafenib, we estimated the IC_50_ values of Caco-2 cells and HT29 cells (both *KRAS*^WT^ and *KRAS*^G12D^). It showed that *KRAS*^G12D^ mutation significantly reduced the sensitivity of CRC cells to regorafenib, as evidenced by markedly increased IC_50_ values in *KRAS*^G12D^ cells compared to their *KRAS*^WT^ counterparts **(**Supplementary Fig. 1E**)**. Given that DHA performed excellently in inhibiting the downstream effects of mutant *KRAS*, we explored whether it could synergize with regorafenib to inhibit CRC. Synergistic anti-proliferative effects were observed across multiple *KRAS*-mutant CRC cell lines, including LS180, SW480, HCT116, and *KRAS*^G12D^-mutant Caco-2 and HT29 cells, upon combination treatment with regorafenib and DHA **(**Fig. 3A, B, Supplementary Fig. 2A–E**)**.

In vitro experiments, including colony formation and apoptosis assay, further revealed that the combination of regorafenib and DHA significantly suppressed the proliferation **(**Fig. 3C, Supplementary Fig. 2F**)** and promoted the apoptosis **(**Fig. 3D, Supplementary Fig. 2G**)** of LS180, SW480 and HCT116 cells compared with either drug alone. For the purpose of uncovering the possible mechanism underlying the synergistic effect, we detected p-Erk1/2 and p-Akt in cell lines. Regorafenib monotherapy had a limited effect on the downregulation of these phosphorylated proteins, whereas its combination with DHA drastically reduced the phosphorylation level **(**Fig. 3E, Supplementary Fig. 2H**)**, indicating enhanced inhibition of the mutant *KRAS* downstream.

To validate the observed synergy in vivo, we established HCT116 xenograft models in mice **(**Supplementary Fig. 3A**)**. At the end of the therapeutic regimen, the mice were sacrificed. Both the tumor size and weight revealed the favorable synergistic effect of regorafenib and DHA on suppressing CRC cell growth **(**Fig. 3F–H**)**. Ki-67 IHC staining and TUNEL assay respectively revealed high efficacy of the combination therapy in inhibiting the proliferation and promoting the apoptosis of CRC cells. And the angiogenesis, as assessed by CD31 expression, was also suppressed **(**Fig. 3I**)**. The body weight fluctuation of mice **(**Supplementary Fig. 3B**)** and H&E staining of liver and kidney **(**Supplementary Fig. 3C**)** indicated no significant adverse effects of the therapeutic combination. Together, these data suggested that DHA could synergized with regorafenib to inhibit CRC by concurrently inhibiting proliferation and promoting apoptosis.

### DHA potentiated regorafenib plus PD-1 monoclonal antibody in *KRAS*-mutant CRCLM

**Fig. 4:**
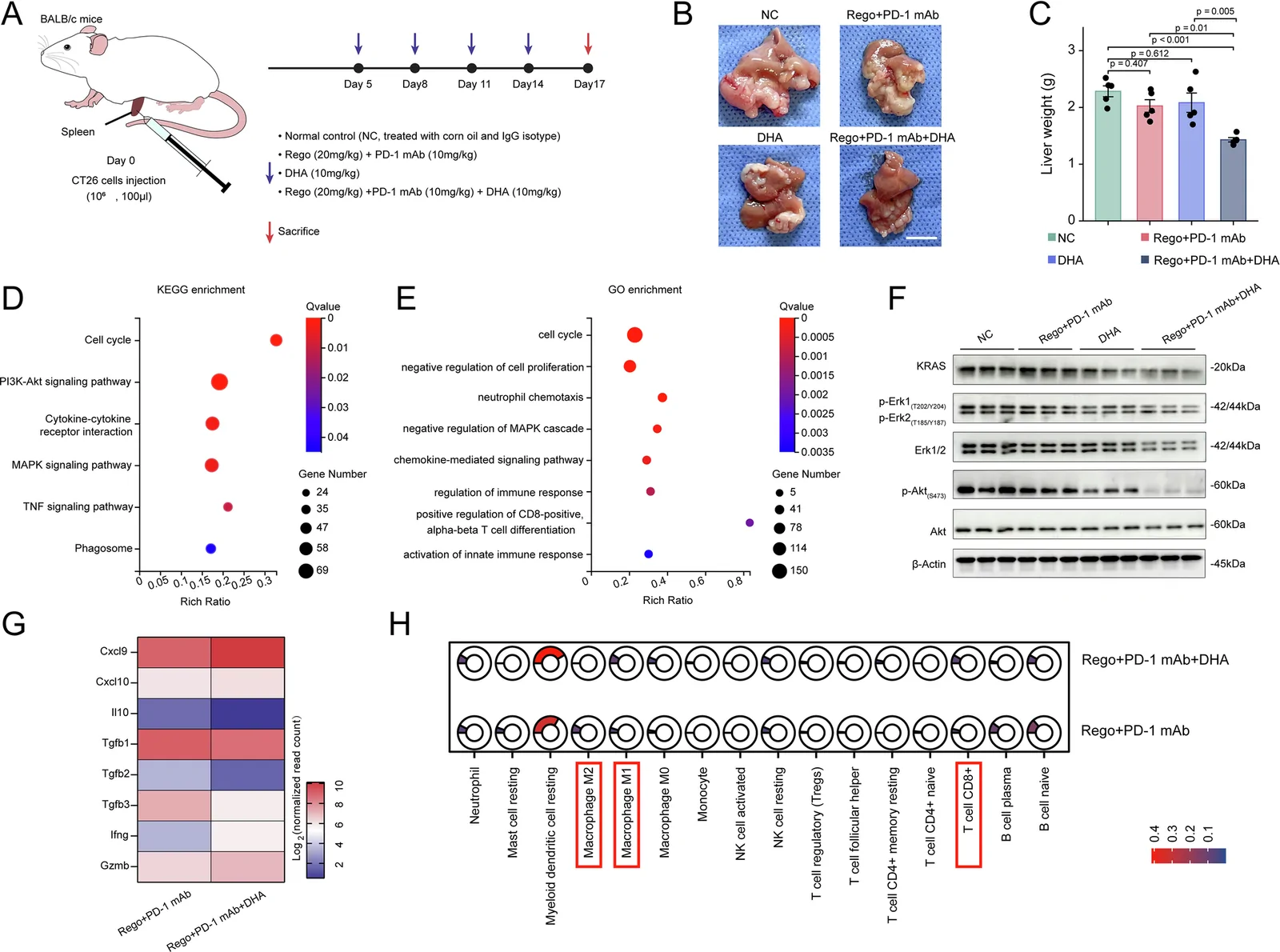
DHA potentiates combinatorial therapy of regorafenib and PD-1 mAb in CRCLM. **A** The experimental workflow of BALB/c mice CRCLM models bearing CT26 cells (*n* = 5 in each group). **B** The liver from BALB/c mice models. **C** The liver weight of BALB/c mice models. **D, E** The KEGG and GO enrichment analyses of bulk RNA-seq conducted with tumor tissues from BALB/c mice models. **F** Western blot showing the downstream signaling pathways (p-Erk1/2 and p-Akt) in mice models tumor tissues. **G** Heatmap showing the immunoregulators expression in tumor tissues based on bulk RNA-Seq. **H** The immune infiltration assessed by CIBERSORT algorithm based on bulk RNA-Seq. NC Normal Control; Rego regorafenib, DHA dihydroartemisinin, mAb monoclonal antibody, KEGG Kyoto Encyclopedia of Genes and Genomes, GO Gene Ontology.

Next, we investigated whether DHA could potentiate the therapeutic efficacy of regorafenib plus PD-1 mAb in the treatment of *KRAS*-mutant CRCLM. To this end, we established CRCLM models by injecting CT26 cells (*Kras*^G12D^) via BALB/c mice spleen, and the therapy went as schedule **(**Fig. 4A**)**. At the experimental endpoint, following euthanasia and tumor excision, we observed that treatment with either the combination of regorafenib and PD-1 mAb, or DHA alone showed limited efficacy. However, when they were combined, tumor suppression was significantly enhanced, indicating a superior therapeutic synergy **(**Fig. 4B, C**)**. Body weight monitoring **(**Supplementary Fig. 3D**)**, and H&E staining **(**Supplementary Fig. 3E**)** indicated no unacceptable adverse effects when the combination of regorafenib, PD-1 mAb and DHA was applied.

To preliminarily explore how DHA enhanced the efficacy of regorafenib and PD-1 mAb combination, we performed bulk RNA-seq on randomly selected samples. The KEGG and GO enrichment analyses suggested that DHA could regulate the proliferation-associated signalings, as well as modulate the anti-tumor immune responses **(**Fig. 4D, E**)**. Consistent with the transcriptomic data, western blot assay confirmed pronounced reductions in KRAS, p-Erk1/2 and p-Akt protein level in the combination with DHA, whereas regorafenib plus PD-1 mAb still inhibited these pathways to a lesser extent in vivo **(**Fig. 4F**)**. Furthermore, DHA treatment led to a decrease in some immunosuppressive factors, like *Tgfb1–3* and *Il-10*, alongside an increase in some immunostimulatory mediators, such as *Cxcl9*, *Cxcl10*, *Gzmb* and *Ifng* **(**Fig. 4G**)**. The TIME landscape depicted by the CIBERSORT algorithm revealed elevated CD8^+^ T cells and M1 macrophages infiltration, and reduced M2 macrophages infiltration **(**Fig. 4H, Supplementary Table 4**)**. These data demonstrated that DHA could substantially augment the anti-CRCLM efficacy of regorafenib plus PD-1 mAb, and hamper the activity of proliferation-related signaling pathways and remodel the TIME.

### ScRNA-seq revealed the effect of DHA on remodeling the TIME in CRCLM

**Fig. 5:**
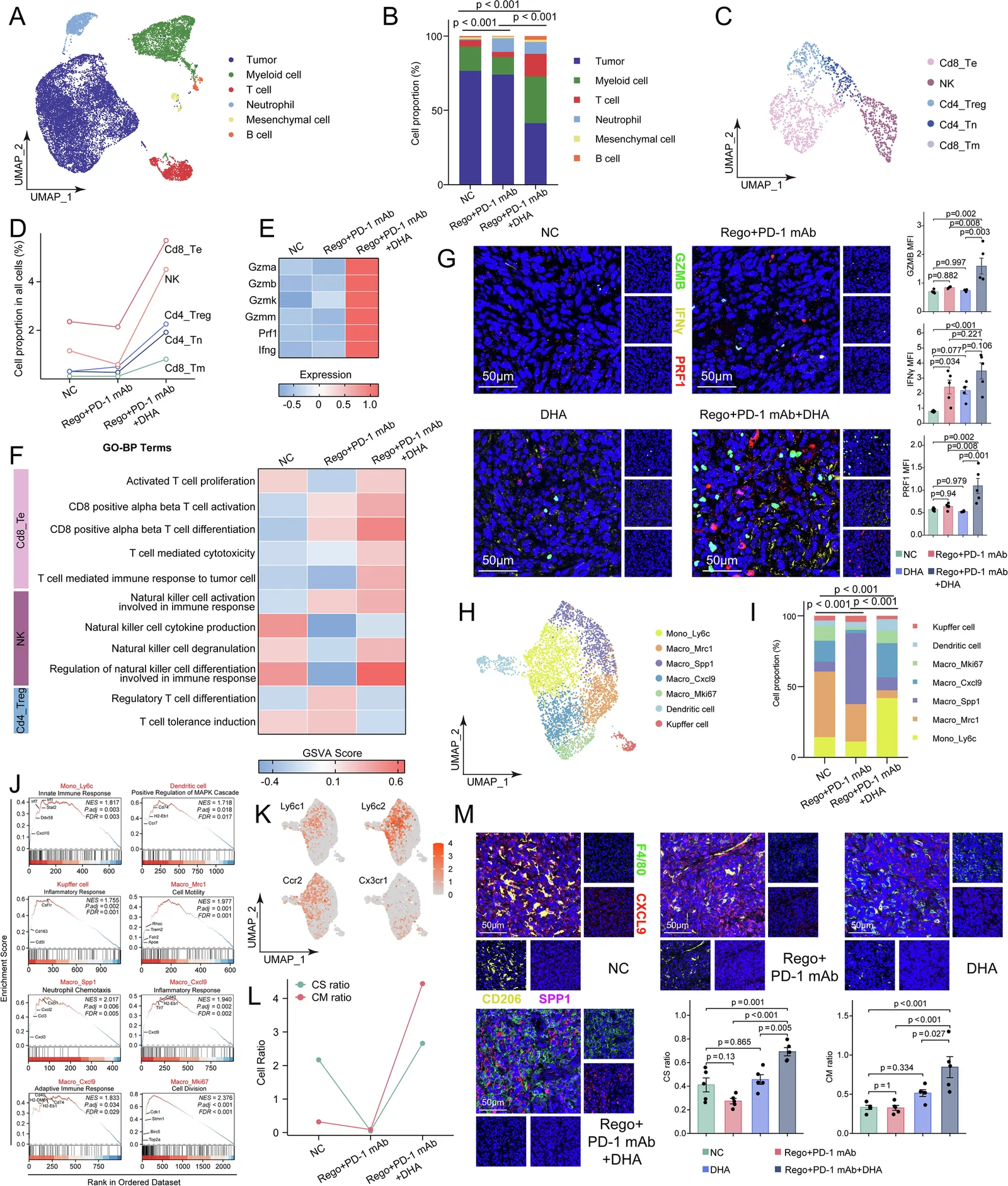
DHA reprograms the TIME in CRCLM. **A** The annotation of main cell types in CRCLM tissues from BALB/c mice models. **B** The proportion of main cell types. **C** The subset of T cells, including Cd8^+^ effector T cells (Cd8_Te), natural killer (NK) cells, Cd4^+^ regulatory T cells (Cd4_Treg), Cd4^+^ naive T cells (Cd4_Tn) and Cd8^+^ memory T cells (Cd8_Tm). **D** The T cells proportion changes in different therapeutic groups. **E** Heatmap showing the cytotoxic chemokines expression based on scRNA-seq. **F** The GSVA of Cd8_Te, NK and Cd4_Treg cells based on scRNA-seq. **G** The validation of T cells cytotoxicity by mIF in situ. **H** The sub-clusters of myeloid cells, including Kupffer cells, dendritic cells, Mki67^+^ macrophages (Macro_Mki67), Cxcl9^+^ macrophages (Macro_Cxcl9), Spp1^+^ macrophages (Macro_Spp1), Mrc1^+^ macrophages (Macro_Mrc1) and Ly6c^+^ monocytes (Mono_Ly6c). **I** The proportion of myeloid cells sub-clusters. **J** The major functional characteristics of each myeloid cells sub-cluster based on GSEA of scRNA-seq. **K** Feature plot showing the phenotype of Mono_Ly6c. **L** The CS ratio and CM ratio in different therapeutic group. **M** Detection of macrophages in tumor tissues from BALB/c mice models in situ. GSVA Gene Set Variation Analysis, GO Gene Ontology, BP Biological Process, MFI Mean Fluorescence Intensity, GSEA Gene Set Enrichment Analysis, NES Normalized Enrichment Score, FDR False Discovery Rate, CS ratio CXCL9/SPP1 ratio, CM ratio CXCL9/MRC1 (CD206) ratio, NC Normal Control, Rego regorafenib, DHA dihydroartemisinin, mAb monoclonal antibody.

In light of the TIME changes suggested by the bulk RNA-seq, we applied scRNA-seq to comprehensively profile the TIME of CRCLM models across different therapeutic groups. Based on the cluster markers **(**Supplementary Fig. 4A**)**, the cells in tumor tissues were categorized into six major populations, including tumor cells, myeloid cells, T cells, neutrophils, mesenchymal cells and B cells **(**Fig. 5A**)**. Notably, DHA treatment led to a dramatic reduction in the proportion of tumor cells, accompanied by a substantial increase in immune cell infiltration, particularly T cells and myeloid cells **(**Fig. 5B**)**. This discrepancy among groups indicated that DHA could drive immune cells chemotaxis and cytotoxicity, thereby potentiating the effect of PD-1 mAb.

Then, for the purpose of dissecting the immune landscape, we subclustered T cells and myeloid cells into distinct functional and phenotypical subsets. T cells comprised Cd8^+^ effector T cells (Cd8_Te), natural killer (NK) cells, Cd4^+^ regulatory T cells (Cd4_Treg), Cd4^+^ naive T cells (Cd4_Tn) and Cd8^+^ memory T cells (Cd8_Tm) **(**Fig. 5C, Supplementary Fig. 4B**)**. The absolute amount of all T cell subtypes increased following DHA treatment, particularly cytotoxic Cd8_Te and NK cells **(**Fig. 5D**)**. Correspondingly, the expression level of cytotoxic factors, such as *Gzma*, *Gzmb*, *Gzmk*, *Gzmm*, *Prf1* and *Ifng*, increased considerably in the group with DHA **(**Fig. 5E**)**. The GSVA revealed that the anti-tumor effects of Cd8_Te and NK cells were enhanced when DHA was included in the therapeutic combination, while the differentiation of Cd4_Treg and its induced tolerance were attenuated **(**Fig. 5F**)**. Moreover, some Cd8_Te and NK cells presented relatively high expression of proliferative markers such as *Mki67*, *Top2a* and *Stmn1*, indicating their potential for amplification to sustain the cytotoxicity **(**Supplementary Fig. 5**)**. These results were validated by the mIF assay, showing an enhanced cytotoxicity by the increased in situ expression of GZMB, IFNγ and PRF1 **(**Fig. 5G**)**.

Myeloid cells, which were clustered into seven subtypes **(**Fig. 5H, Supplementary Fig. 4C**)**, also manifested distinct shifts in response to different treatments. In the DHA-contained combination-treated group, the proportions of Ly6c^+^ monocytes (Mono_Ly6c) and Cxcl9^+^ macrophages (Macro_Cxcl9, identified as pro-inflammation types) were markedly increased, while the proportion of anti-inflammation macrophage subsets, like Mrc1^+^ macrophages (Macro_Mrc1) and Spp1^+^ macrophages (Macro_Spp1) were significantly decreased **(**Fig. 5I**)**. GSEA revealed the characteristics of each subtype and further confirmed the precision of subclustering **(**Supplementary Fig. 6A–G**)**. Mono_Ly6c functioned to activate the innate immune response **(**Fig. 5J**)**, and phenotypically, it was a cluster of Ly6c1^+^, Ly6c2^+^, Ccr2^+^ and Cx3cr1^-^ cells **(**Fig. 5K**)**, which was reported as a type of pro-inflammation monocytes [25]. Mrc1, also known as CD206, is a representative marker of M2 macrophages. The Macro_Mrc1 manifested a gene signature of cell motility, which was consistent with the findings of a previous study revealing that the contractility within the actin cytoskeleton contributed to the M2 polarization of macrophages [26]. Another type of anti-inflammation macrophage, Macro_Spp1, showed a characteristic of neutrophil chemotaxis **(**Fig. 5J**)**. In contrast, the pro-inflammation macrophage, Macro_Cxcl9, was associated with inflammation response and adaptive immune response **(**Fig. 5J**)**. A recent study has defined macrophage polarization based on CXCL9 and SPP1 expression (CS) ratio [27]; that is, a high CS ratio indicates polarization to M1-like macrophages. Concordantly, in our analyses, CS ratio, as well as the Cxcl9 and Mrc1 expression (CM) ratio, was elevated in the group treated with DHA combined **(**Fig. 5L, Supplementary Fig. 6H**)**. These results were also corroborated with mIF staining in situ, which further validated the role of DHA in promoting the M1 type polarization of macrophages in CRCLM **(**Fig. 5M**)**.

In addition to macrophages and monocytes, neutrophils are also derived from myeloid progenitor cells, and we also analyzed their changes. Although the ratio of neutrophils declined slightly with DHA treatment **(**Fig. 5B**)**, the Irf1^+^ and Cd74^+^ neutrophils, which have been proved important in ICB therapy [28, 29], showed noticeable increases **(**Supplementary Fig. 6I–K**)**. GSVA displayed the elevated anti-tumor activity, such as antigen presentation and regulation of T cell-mediated immune response. Besides, programmed cell death in response to reactive oxygen species was downregulated and apoptotic chromosome condensation was upregulated **(**Supplementary Fig. 6L**)**. Collectively, these results demonstrated that DHA exerted profound modulatory effects on the TIME, promoting cytotoxic and pro-inflammatory immune cell populations, while suppressing immunosuppressive subsets.

### DHA activated the interferon response signaling to regulate the immune responses

**Fig. 6:**
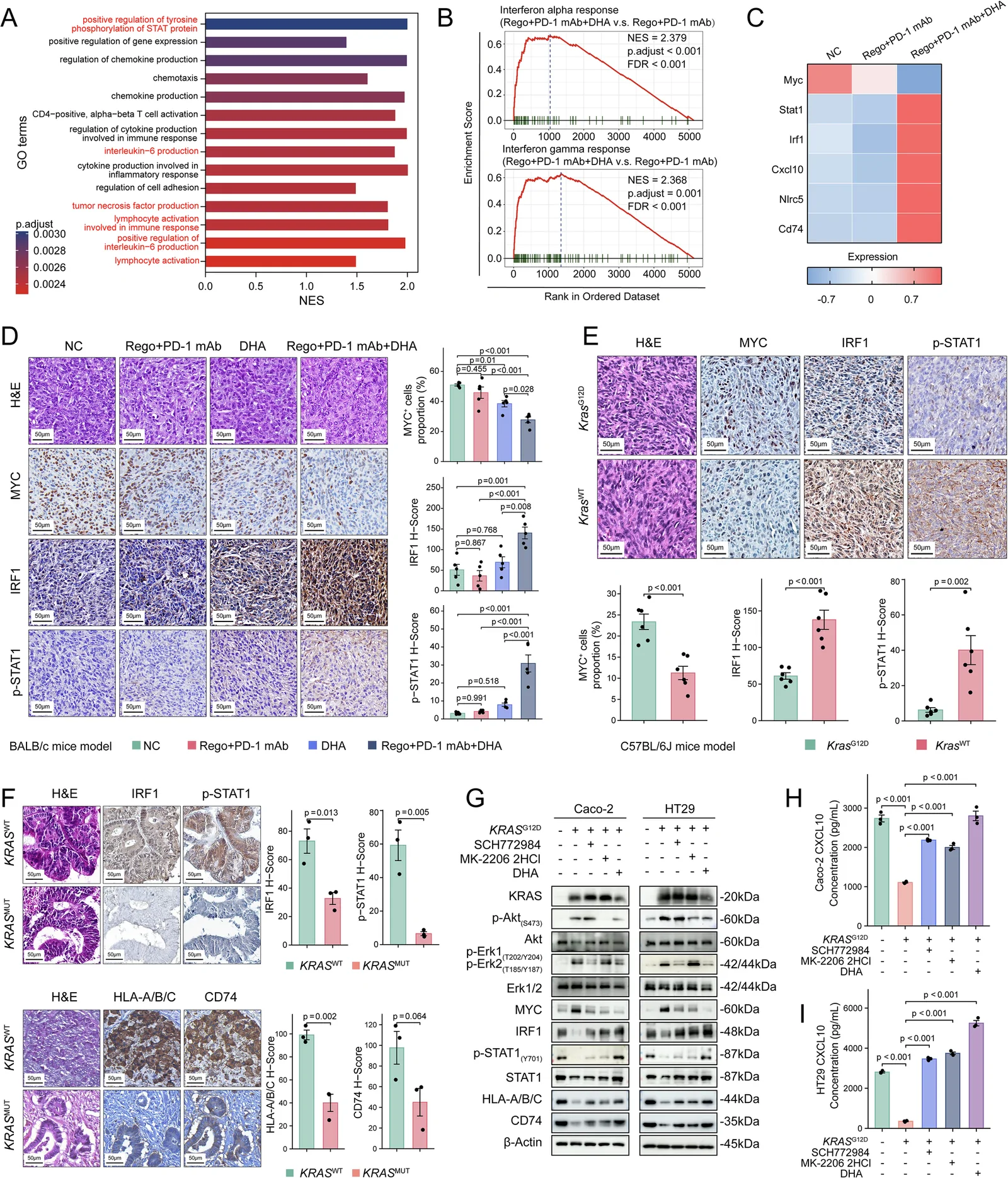
DHA reshapes T cells immune response via restoring IFN response inhibited by mutant KRAS in CRC cells. **A** GSEA of tumor cells between Rego+PD-1 mAb+DHA and Rego+PD-1 mAb group. **B** IFNα and IFNγ response enrichment in tumor cells between Rego+PD-1 mAb group and Rego+PD-1 mAb+DHA group based on scRNA-seq. **C** Heatmap showing IFN response-associated genes expression in tumor cells. **D** IHC staining of MYC, IRF1 and p-STAT1 in tumor tissues of BALB/c mice models (*n* = 5 in each group). **E** IHC staining of MYC, IRF1 and p-STAT1 in tumor tissues of C57BL/6 J mice models (*n* = 6 in each group). **F** IHC staining of IRF1, p-STAT1, HLA-A/B/C and CD74 in clinical samples (*n* = 3 in each group). **G** Western blot showing IFN response-associated genes expression in Caco-2 and HT29 cells with or without *KRAS*^G12D^ and treated with p-Erk inhibitor (SCH772984), p-Akt inhibitor (MK-2206 2HCl) or DHA. **H, I** CXCL10 concentration in the supernatant of Caco-2 and HT29 cells detected by ELISA. The supernatant was diluted 20-fold before assessment (*n* = 3 replicates). GSEA Gene Set Enrichment Analysis, NES Normalized Enrichment Score, GO Gene Ontology, NC Normal Control, Rego regorafenib, DHA dihydroartemisinin, mAb monoclonal antibody.

Since DHA could inhibit the expression of mutant KRAS and remodel the TIME of CRCLM to enhance the efficacy of PD-1 mAb, we then tried to elucidate the underlying mechanism of KRAS regulating the anti-tumor immune response. GSEA showed differences in the activities of several immune-related biological processes between groups treated with or without DHA combined **(**Fig. 6A**)**, which was associated to IFN response, and differences of protein tyrosine kinase activity between normal control and regorafenib plus PD-1 mAb group, which has been preliminarily verified (Supplementary Fig. 8**)** and interpreted in Discussion section. We then analyzed the activity of IFN response, and found that it was upregulated in the group treated with regorafenib, PD-1 mAb and DHA, compared with DHA-absent group **(**Fig. 6B**)**. The expression of some effective genes involved in IFN response, such as *Stat1*, *Irf1*, *Cxcl10*, *Nlrc5* and *Cd74*, was increased, while the expression of *Myc*, a negative regulator of IFN response, was reduced **(**Fig. 6C**)**, which was partially validated by IHC staining in BALB/c mice tumor tissues **(**Fig. 6D**)**. In C57BL/6 J mice models, the *Kras*^G12D^ tumors had lower expression of IRF1 and p-STAT1, and higher expression of MYC **(**Fig. 6E**)**, and in clinical samples, tumor with mutant *KRAS* had lower IRF1, p-STAT1, HLA-A/B/C (downstream of NLRC5 [30], and both HLA-A/B/C and NLRC5 can be promoted by key transcription factors, IRF1 and p-STAT1, in IFN response [31]) and CD74 expression **(**Fig. 6F**)**, which further verified our findings from scRNA-seq analysis.

It has been proven that p-Erk1/2 and p-Akt, the downstream signalings of *KRAS*, could suppress IFN response via upregulating *Myc* in lung cancer [32]. In order to validate this mechanism in CRCLM, we used p-Erk1/2 and p-Akt inhibitors in *KRAS*^G12D^ Caco-2 and HT29 cells. As was expected, the inhibition of p-Erk1/2 and p-Akt elevated IFN response, and DHA also worked by concurrently inhibiting p-Erk1/2 and p-Akt **(**Fig. 6G–I**)**.

**Fig. 7:**
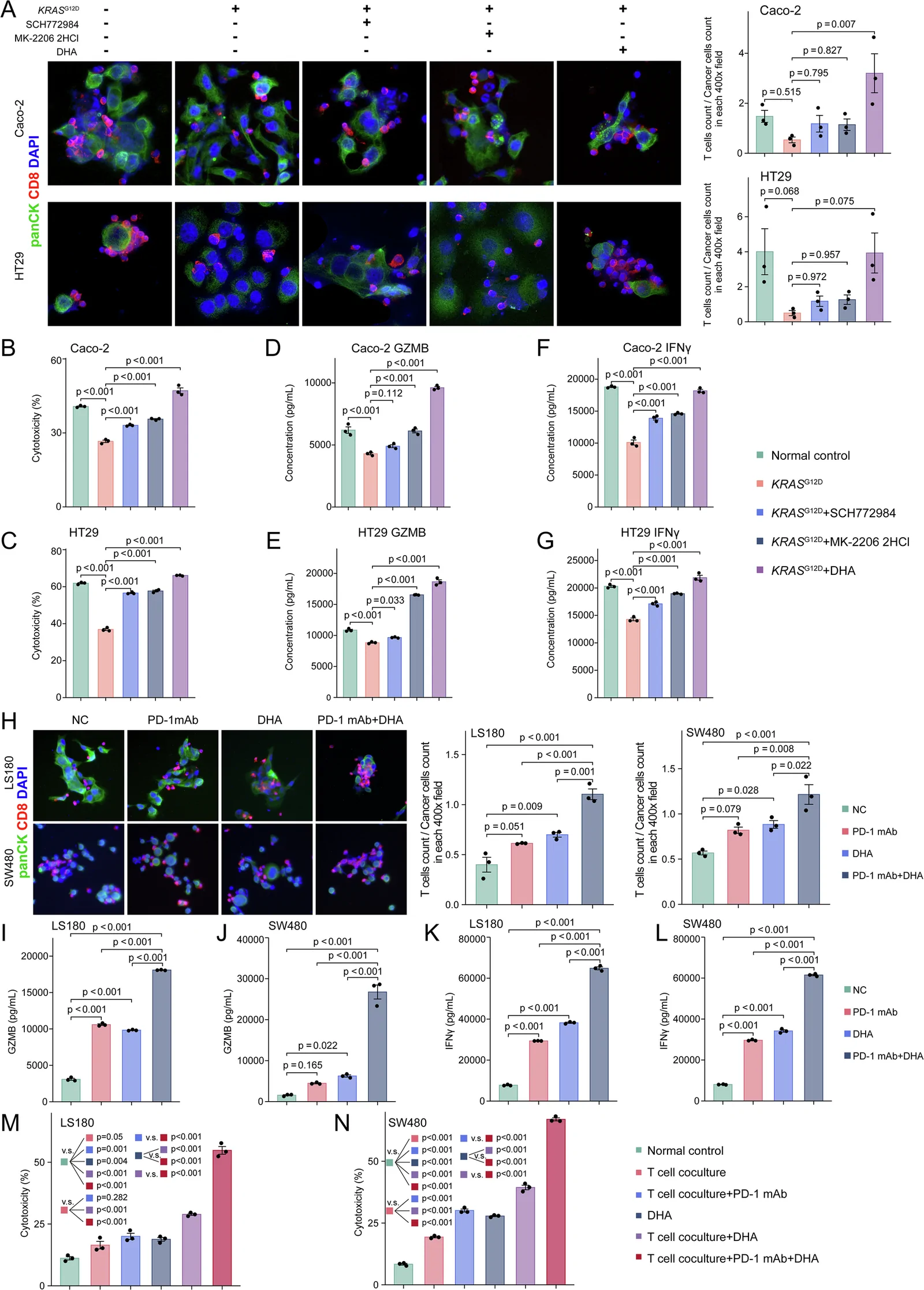
DHA promotes the chemotaxis and cytotoxicity of CD8^+^ T cells in CRC by inhibiting KRAS, thus synergizing with PD-1 mAb. **A** The cellular immunofluorescence of the co-culture system containing Caco-2 or HT29 cells and CD8^+^ T cells (*n* = 3 replicates). The co-coculture lasted for 24 h. T cells and cancer cells ratio per 400× field is regarded to define the chemotaxis of T cells. **B, C** The cytotoxicity assessed by LDH in co-culture system (*n* = 3 replicates). **D, E** GZMB concentration in the supernatant of co-culture system detected by ELISA (*n* = 3 replicates). The supernatant was diluted 50-fold before assessment. **F**, **G** IFNγ concentration in the supernatant of co-culture system detected by ELISA (*n* = 3 replicates). The supernatant was diluted 50-fold before assessment. **H** The cellular immunofluorescence of the co-culture system containing LS180 or SW480 cells and CD8^+^ T cells (*n* = 3 replicates). The co-coculture lasted for 24 h. T cells and cancer cells ratio per 400× field is regarded to define the chemotaxis of T cells. **I, J** GZMB concentration in the supernatant of co-culture system detected by ELISA (*n* = 3 replicates). The supernatant was diluted 50-fold before assessment. **K**, **L** IFNγ concentration in the supernatant of co-culture system detected by ELISA (*n* = 3 replicates). The supernatant was diluted 50-fold before assessment. **M, N** The cytotoxicity assessed by LDH in co-culture system (*n* = 3 replicates). DHA dihydroartemisinin, LDH lactate dehydrogenase.

Among the effective factors of IFN response, CXCL10 is a chemokine that binds to CXCR3 on T cells to promote T cell migration [33], and HLA-A/B/C and CD74 play important role in presenting antigens to T cells [30, 34]. Therefore, we co-cultured CD8^+^ T cells sorted from human PBLCs **(**Supplementary Fig. 7**)** and cancer cells treated with p-Erk1/2 or p-Akt inhibitor or DHA to further investigate the effect of IFN response of cancer cells on T cells. In the co-culture system, the *KRAS*^G12D^ cancer cells inhibited the chemotaxis **(**Fig. 7A**)**, and the cytotoxicity **(**Fig. 7B, C**)** of CD8^+^ T cells, along with reduced GZMB and IFNγ **(**Fig. 7D–G**)**, while inhibition of p-Erk1/2 and p-Akt with specific inhibitors or DHA reversed the immune escape.

Given that DHA could promote CD8^+^ T cell-mediated immunity by promoting chemotaxis and cytotoxicity, we applied the co-culture system to investigate whether it could synergize with PD-1 mAb. It showed that, in the co-culture systems with LS180 cells or SW480 cells, DHA was able to synergize with PD-1 mAb to promote CD8^+^ T cells chemotaxis **(**Fig. 7H**)** and cytotoxicity **(**Fig. 7I–N**)**. These data suggested that DHA could attenuate the immune escape by restoring IFN response via robustly inhibiting KRAS^G12D^ expression.

## Discussion

Various types of cancers, including CRCLM, present a highly suppressive TIME, commonly referred to as a “cold tumor”, where there is a lack of target for ICB. Therefore, shifting a “cold tumor” to a “hot tumor” is essential to sensitize patients to ICB therapy. Targeted therapy combined with ICB is exactly such an attempt, wherein targeted therapy reduces tumor burden to restrain the suppressive microenvironment and ICB reactivates immune response. However, we observed resistance to this combinatorial regimen in our clinical practices. Although small amount of CRCLM cases were enrolled for analyses because the patients with advanced-stage diseases don’t commonly accept surgery and their specimens are difficult to obtain, we used mice models to certify the role of mutant *KRAS* in impairing the efficacy of regorafenib plus anti-PD-1 in CRCLM. We also identified DHA as a potential mutant KRAS inhibitor by effectively downregulating its expression, and most importantly, DHA could potentiate regorafenib plus PD-1 mAb via restoring the activity of IFN response.

In the drug virtual screening, DHA ranked third, following andrographolide and allantoin. Since andrographolide has not been reported to exert favorable immunoregulatory effects in CRC and has a very low bioavailability [35, 36], and allantoin is primarily used for dermatological conditions, DHA was selected for the subsequent investigations. Molecular docking, molecular dynamics simulation and SPR assay of DHA-KRAS^G12D^ complex validated the favorable binding affinity between them. And functionally, DHA performed excellently in inhibiting KRAS^G12D^ expression, potentiating the therapeutic efficacy of regorafenib and PD-1 mAb, and exhibited few adverse effects, such as weight loss, liver and kidney histopathologic toxicity. Additionally, DHA appeared to able to downregulate multiple mutant KRAS, KRAS^G12D^, KRAS^G12V^ and KRAS^G13D^ in this study, which implies that it could potentially address the secondary KRAS mutation that emerges during treatment. Moreover, we have preliminarily revealed that DHA downregulates KRAS^G12D^ expression by promoting autophagy-lysosome pathway, and Ser-145 was a key residue in DHA-induced KRAS^G12D^ downregulation. Ser145 is one of the amino acid residues constituting the nucleobase-binding loop of KRAS protein and belongs to the β6 region, which is critical for the GTPase activity of KRAS. Mutations G12D and G13D can perturb the hydrogen-bonding network in the β6 region and thereby impairing its enzymatic activity [37]. In the present study, enzymatic activity was not assayed because DHA directly downregulated the protein level of mutant KRAS, thus abrogating their function. On the other hand, the local structure of the β6 region in wild-type KRAS differs from that in the G12D and G13D mutants, which may prevent DHA from binding efficiently to this region and can explain why DHA did not significantly affect the expression level of wild-type KRAS. Nevertheless, the current results only demonstrate that the Ser-145 residue is essential for DHA-induced degradation of KRAS^G12D^. Whether this residue serves as a direct binding site for DHA, or whether it is equally important for the degradation of other KRAS mutants, remains to be further investigated. Notwithstanding this, based on the current evaluation of the efficacy and safety of the combination therapy regimen, as a clinically approved drug, DHA still holds great promise to become a component of combination therapies in the future. These findings offer new treatment options for CRCLM and even other cancers driven by *KRAS* mutations.

The scRNA-seq revealed that DHA exerted a profound influence on remodeling TIME, including enhancing antigen presentation, promoting T cells chemotaxis and cytotoxicity, and driving macrophages polarization toward the pro-inflammatory phenotype. Mechanically, DHA can activate IFN response in cancer cells by inhibiting mutant KRAS that can promote Akt/Erk-MYC signaling [38, 39]. MYC has been reported as an inhibitor of IFN response [40, 41], and we primarily detected the expression of MYC in both tumor tissues and cell lines to make a pathway-consistent interpretation. The pivotal role of IFN response in shaping TIME and regulating the efficacy of ICB therapy has been extensively investigated, indicating that the downregulation of IFN response by various factors can attenuate anti-tumor immune response and diminish ICB therapy efficacy [42–44]. Consistent with our findings, a previous study showed that inhibiting KRAS^G12C^ in lung cancer could also enhance IFN response to promote anti-tumor immune response [32]. These studies indicate that *KRAS* mutation can suppress IFN response in cancer cells to facilitate immune evasion, and that the mutant KRAS is a critical target for DHA to remodel the TIME. The CD8^+^ T cells and cancer cells co-culture system was used for validating the impact of KRAS mutation-induced low IFN response on T cells function, which further supports the underlying mechanism that the diminished IFN response in cancer cells can impair T cells chemotaxis and cytotoxicity. In the co-culture experiments, tumor cells and T cells were co-cultured for only 24 h, rather than 48 h as used in the previous protein detection assays **(**Fig. 6**)**. This shorter incubation period was adopted to avoid excessive tumor cell death caused by prolonged co-culture, which would lead to inaccurate assessment of T cells chemotaxis. This may explain why inhibition of p-Akt and p-Erk1/2 showed only a trend toward promoting chemotaxis without reaching statistical significance. Nevertheless, DHA still significantly enhanced T cells chemotaxis at the same incubation time. Although the precise mechanisms by which DHA regulated macrophages polarization were not fully investigated in this study, the GSEA suggested that adding DHA in the combination therapy could promote the production of tumor necrosis factor, which has been reported as an M1 macrophage polarization mediator [45]. Some studies have also reported that DHA can be taken up by macrophages and may promote M1 polarization [46]. In the tumors treated with regorafenib, PD-1 mAb, and DHA, the proportion of Mki67^+^ macrophages was higher than that in the other two groups. Consistently, GSEA indicated that this Mki67^+^ macrophage cluster exhibited a proliferation-associated transcriptional profile. Therefore, this cluster may represent an expanded macrophage pool in the TIME, which can be further shaped toward a pro-inflammatory/M1-like state in response to DHA. However, given the intricacy of macrophage polarization regulation, additional studies are warranted to clarify the precise mechanisms underlying the modulation of macrophage polarization by DHA. The present study also suggested that DHA could potentially regulated the phenotypes of neutrophils in CRCLM, among which the downregulated programmed cell death in response to reactive oxygen species and upregulated apoptotic chromosome condensation were related to neutrophil extracellular traps formation [47].

Targeting mutant KRAS or the downstream signaling pathways can induce resistance through multiple mechanisms, including reactivation of the signal cascade [19, 20, 48] and activation of parallel signaling pathways [49, 50]. Here, in our study, regorafenib is a broad-spectrum tyrosine kinase inhibitor, which can inhibit the downstream signalings of KRAS, such as p-Akt and p-Erk1/2. However, regorafenib showed little effect in the combination with PD-1 mAb. The scRNA-seq revealed that protein tyrosine kinase activity was upregulated in the combination-treated group, in which *Areg*, *Ereg* and *Hbegf*, the ligands of ERBB receptors, were enriched and upregulated, and GSVA presented the similar situation **(**Supplementary Fig. 8A–C**)**. The IHC staining of tumor tissues from BALB/c mice models validated the results of scRNA-seq **(**Supplementary Fig. 8D**)**. Given that the mutant *KRAS* can usually lead to drug resistance when its signaling being targeted, we explored whether the activation of ERBB signaling pathway induced by regorafenib relies on the presence of mutant *KRAS*. It showed that the ERBB ligands could be upregulated in *KRAS*^G12D^ CRC cells with regorafenib administrated in a long-term (96 h) **(**Supplementary Fig. 8E**)**, which was similar to the IHC staining in tumor tissues from C57BL/6 J mice models **(**Supplementary Fig. 8F**)**. P-EGFR, one of the ERBB receptors, was less inhibited by regorafenib in *KRAS*^G12D^ CRC cells **(**Supplementary Fig. 8G**)**. Therefore, the cells harboring *KRAS*^G12D^ basically showed higher activation of ERBB signaling. These preliminary findings were similar with previous studies that revealed parallel signalings causing drug resistance. To reverse the acquired drug resistance, the co-inhibition of KRAS and EGFR has been evaluated in some preclinical studies and clinical trials [51–53], demonstrating effective tumor suppression. However, in *KRAS*-mutant cancer, the drug resistance can also be induced by some other mechanisms, for example, the secondary mutation of *KRAS* [54, 55]. Under the present circumstance of the scarcity of KRAS inhibitors, depleting the function of mutant KRAS to counteract tumor progression and drug resistance is still a formidable challenge. And here, although we did not do further exploration of the activation of ERBB by regorafenib treatment, the DHA did show great efficacy in assisting regorafenib to inhibit tyrosine kinase and reduce ERBB activation **(**Supplementary Fig. 8E, G**)**.

In summary, our study validates *KRAS* mutation mediates resistance to regorafenib plus anti-PD-1 therapy in CRCLM, which is probably caused by the activation of ERBB signaling pathway. We further demonstrated that DHA, a clinically approved drug, functions as a multi-KRAS mutant inhibitor capable of suppressing KRAS mutant protein expression, restoring IFN response, and remodeling the TIME. Notably, DHA enhances the efficacy of regorafenib combined with PD-1 blockade, while exhibiting no significant adverse effects. These findings provide a compelling rationale for incorporating DHA into combination regimens for the treatment of CRCLM and potentially other *KRAS*-mutant malignancies. Moving forward, deeper investigations will be essential to facilitate the clinical translation of DHA-based therapeutic strategies and advance the development of precision medicine approaches to overcome drug resistance in cancer therapy.

## Supplementary information

Supplementary Files

WB full gels and blots

## Data Availability

The research data related to patients are confidential based on hospital-relevant policies and regulations to protect patients’ information security and privacy. The data from animals and cell lines are available upon reasonable request.
