# Supplementary material for: Dihydroartemisinin inhibits mutant KRAS to potentiate regorafenib plus anti-PD-1 in KRAS-mutant colorectal cancer liver metastases
Source: Cell Death Dis. 2026 Jul 6;17(1):831. doi: 10.1038/s41419-026-09042-z (PMC13612556; doi:10.1038/s41419-026-09042-z)

Fig. 1G

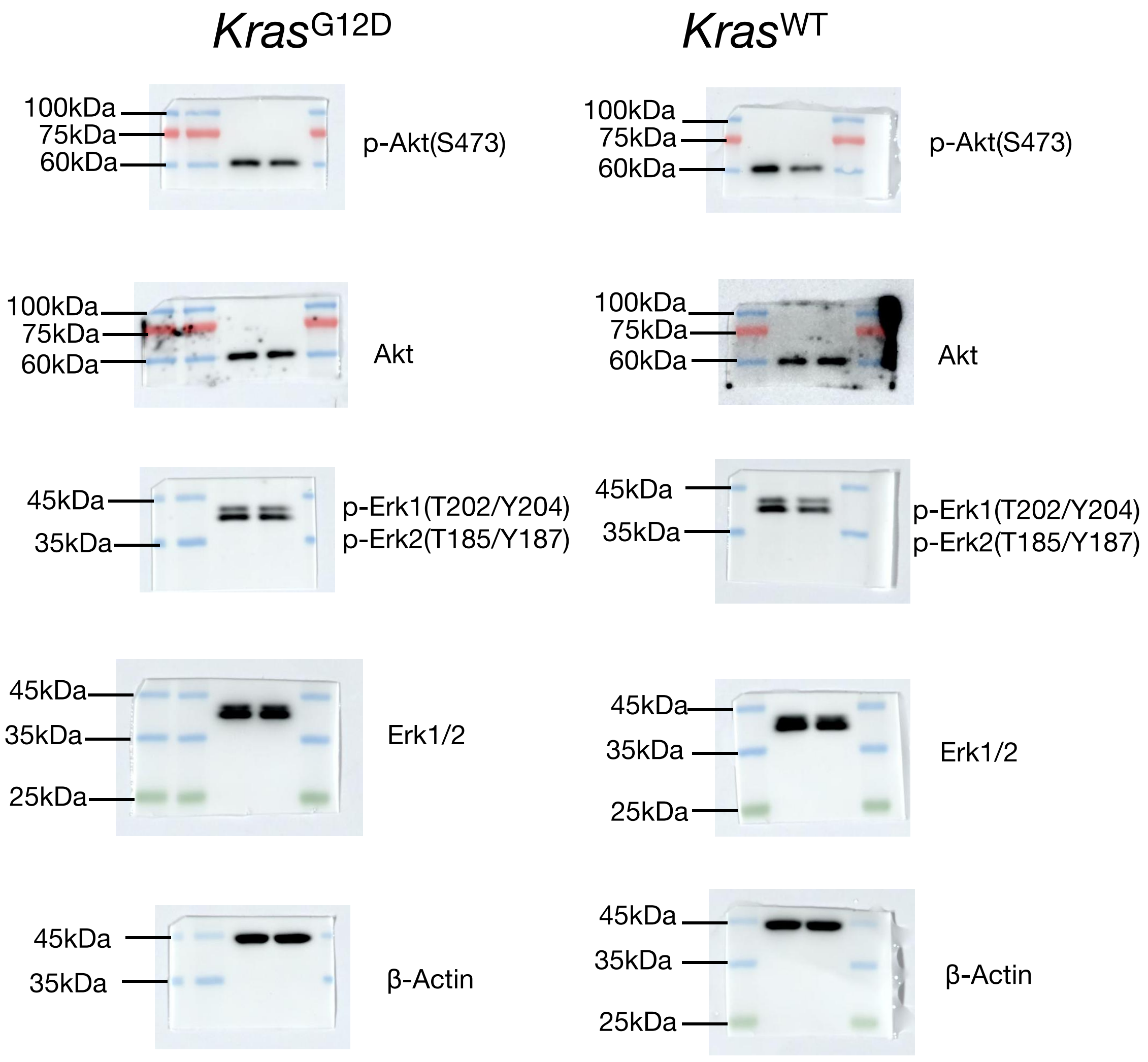

KrasG12D
KrasWT
100kDa
75kDa
60kDa
p-Akt(S473)
Akt
45kDa
35kDa
25kDa
p-Erk1(T202/Y204)
p-Erk2(T185/Y187)
Erk1/2
β-Actin

Fig. 2F

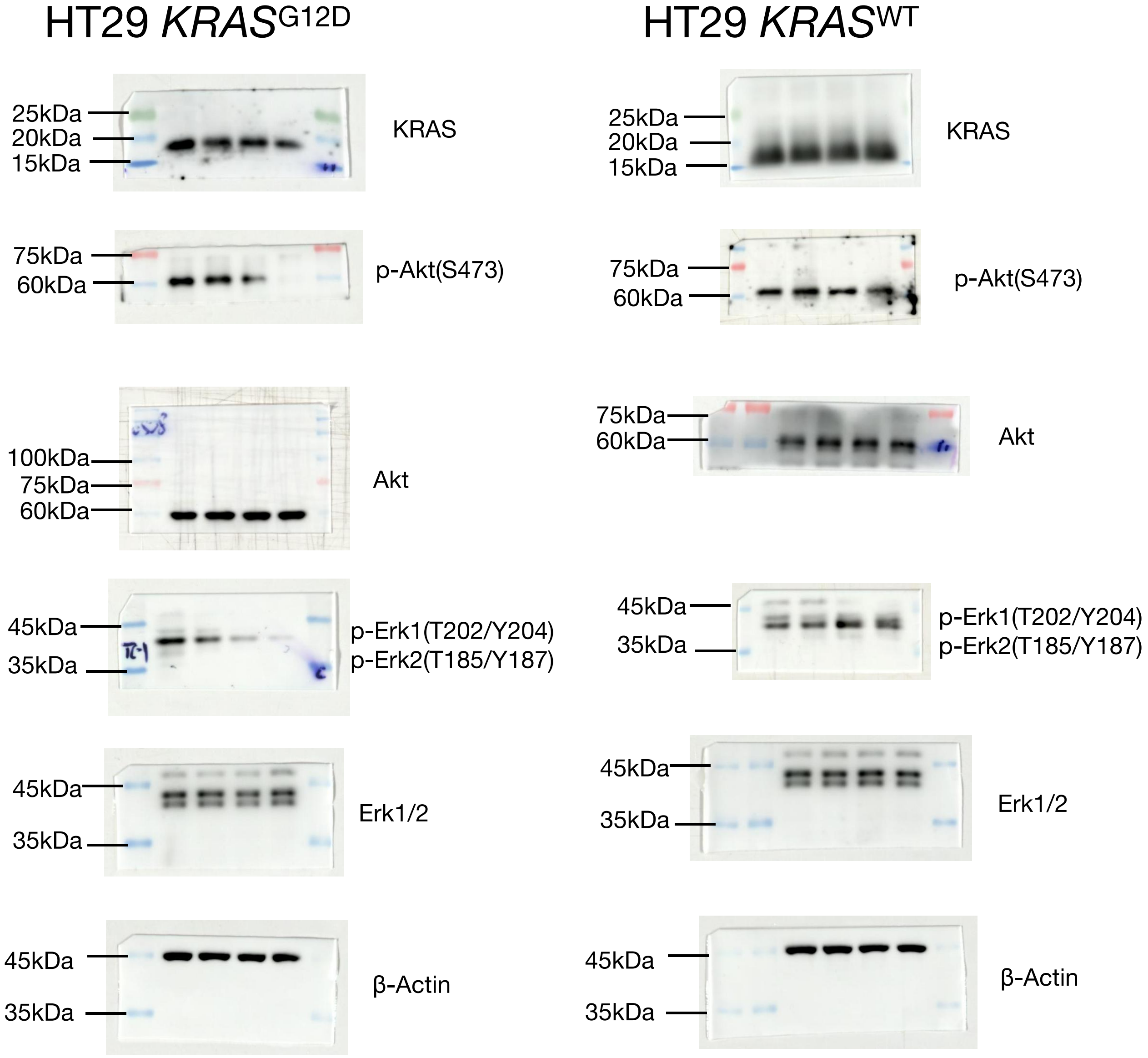

HT29 KRASG12D
HT29 KRASWT
25kDa
20kDa
15kDa
KRAS
75kDa
60kDa
p-Akt(S473)
100kDa
Akt
45kDa
35kDa
p-Erk1(T202/Y204)
p-Erk2(T185/Y187)
Erk1/2
β-Actin

Fig. 2F

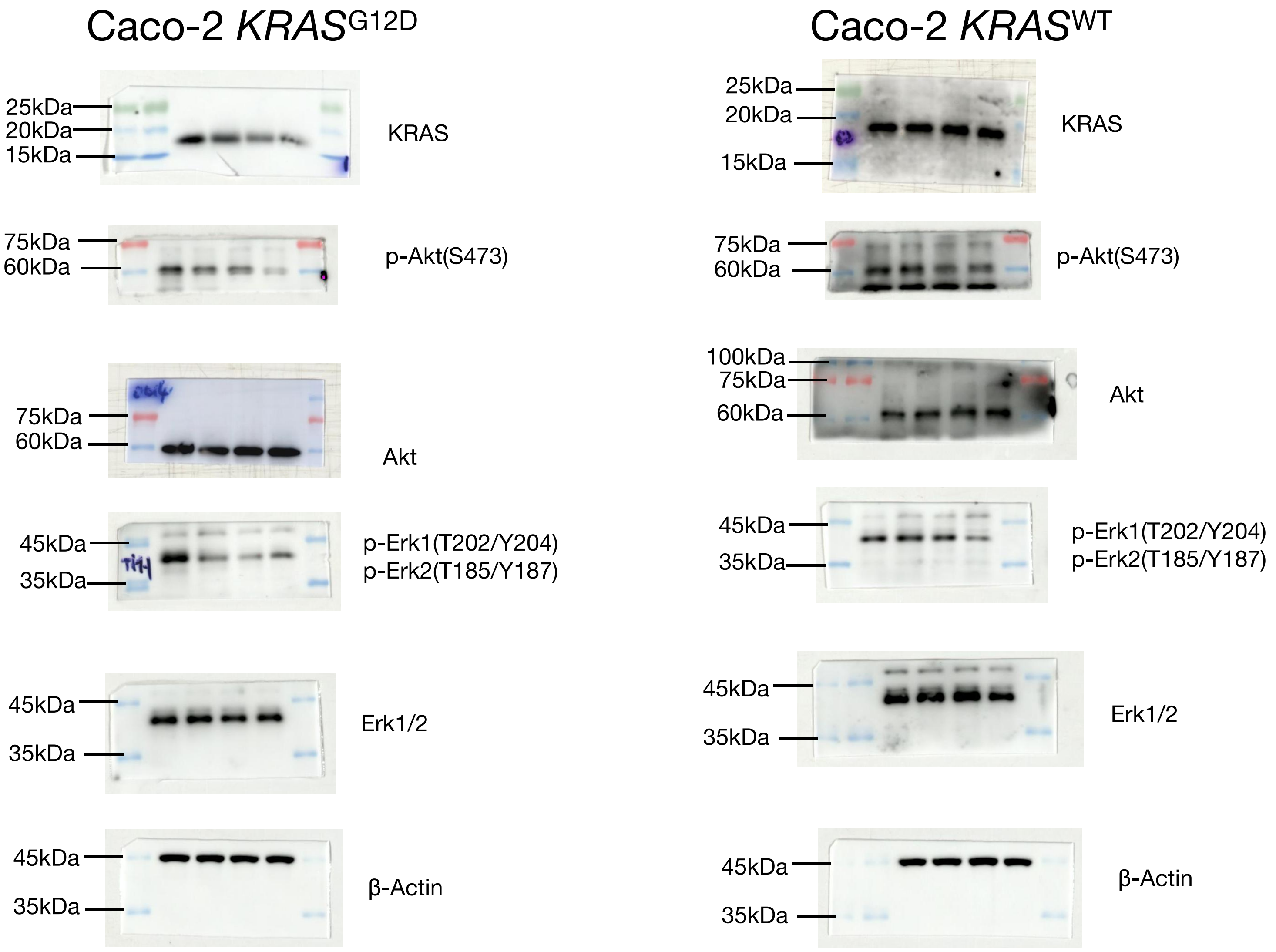
Caco-2 *KRAS*G12D
25kDa
20kDa
15kDa
KRAS
75kDa
60kDa
p-Akt(S473)
75kDa
60kDa
Akt
45kDa
35kDa
p-Erk1(T202/Y204)
p-Erk2(T185/Y187)
45kDa
35kDa
Erk1/2
45kDa
35kDa
β-Actin
Caco-2 *KRAS*WT
25kDa
20kDa
15kDa
KRAS
75kDa
60kDa
p-Akt(S473)
100kDa
75kDa
60kDa
Akt
45kDa
35kDa
p-Erk1(T202/Y204)
p-Erk2(T185/Y187)
45kDa
35kDa
Erk1/2
45kDa
35kDa
β-Actin

Fig. 2F

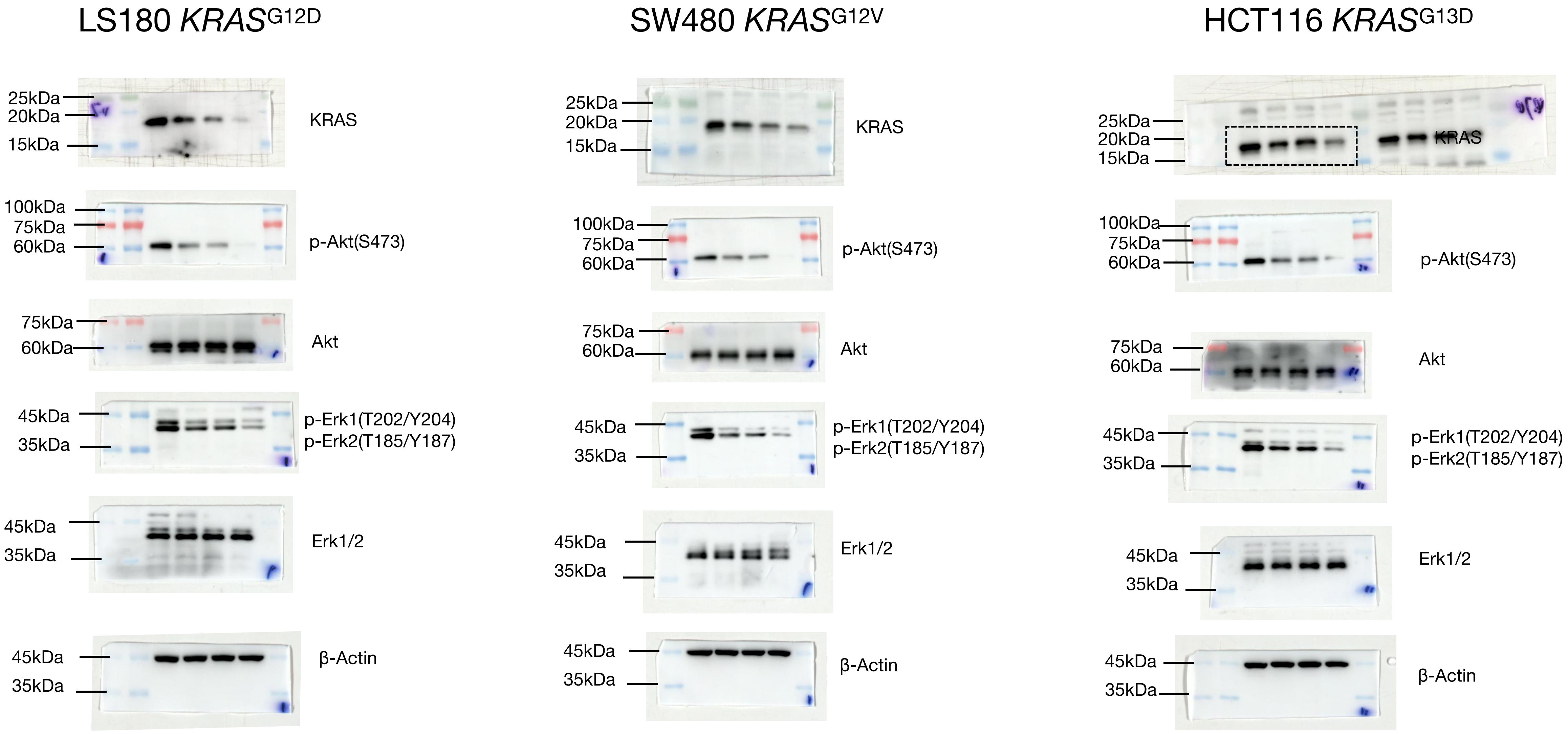

LS180 KRAS G12D
SW480 KRAS G12V
HCT116 KRAS G13D
25kDa
20kDa
15kDa
KRAS
100kDa
75kDa
60kDa
p-Akt(S473)
75kDa
60kDa
Akt
45kDa
35kDa
p-Erk1(T202/Y204)
p-Erk2(T185/Y187)
45kDa
35kDa
Erk1/2
45kDa
35kDa
β-Actin

Fig. 2G

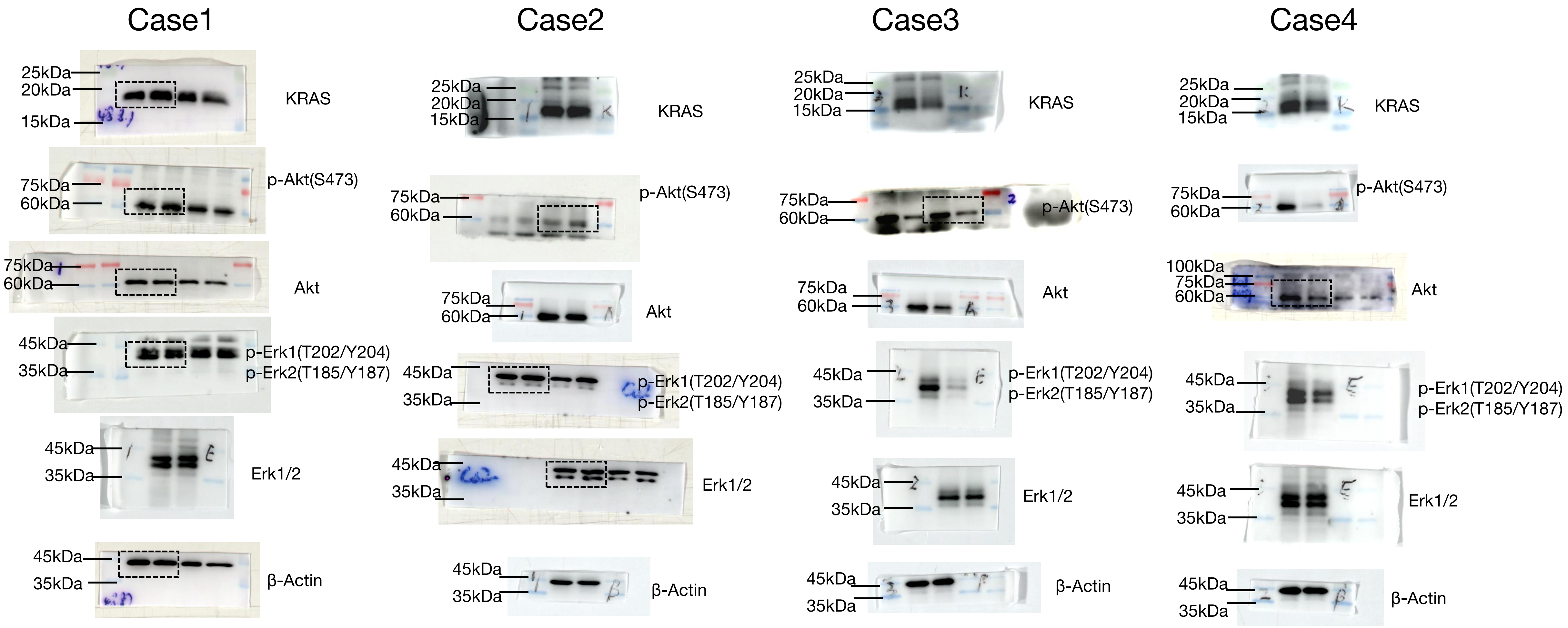
Case1
Case2
Case3
Case4
25kDa
20kDa
15kDa
KRAS
75kDa
60kDa
p-Akt(S473)
Akt
100kDa
45kDa
35kDa
p-Erk1(T202/Y204)
p-Erk2(T185/Y187)
Erk1/2
β-Actin

Fig. 2H

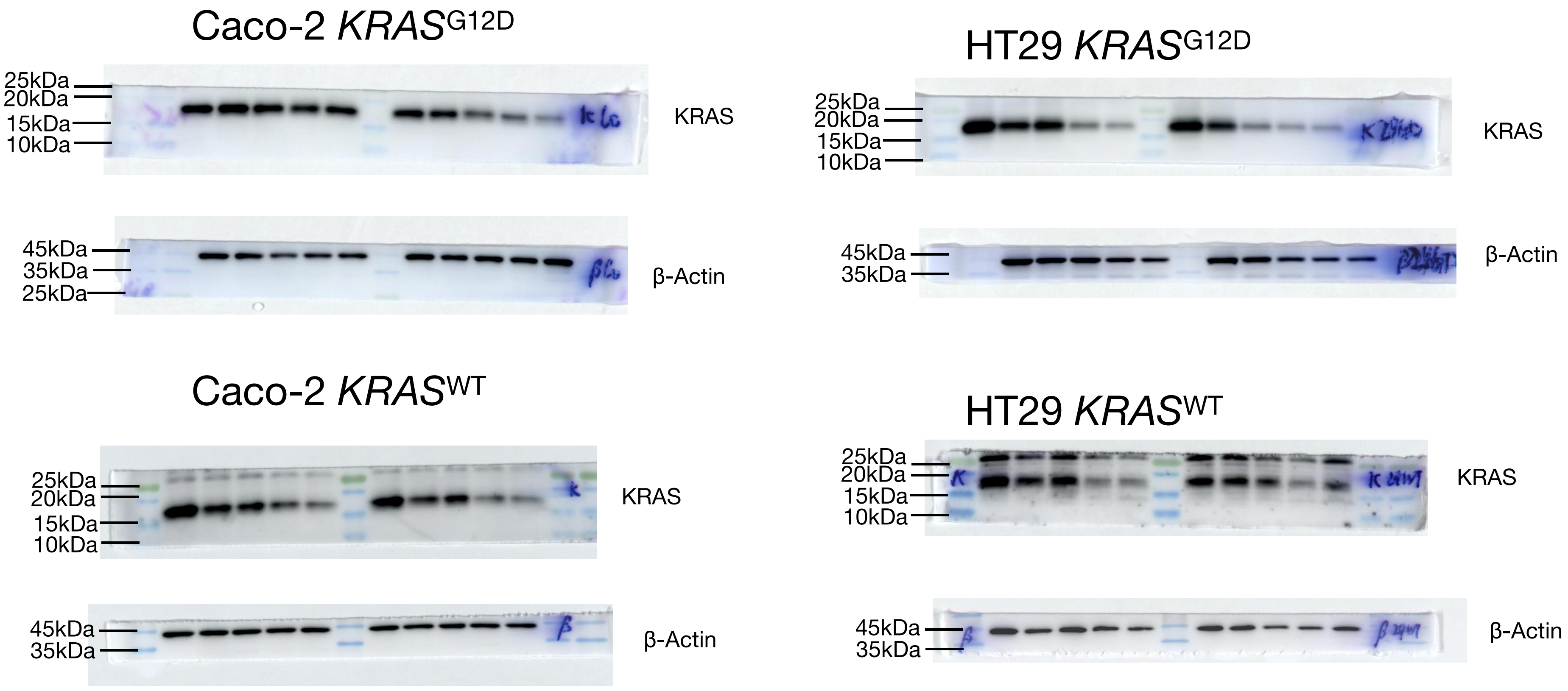
Caco-2 $KRAS^{G12D}$
25kDa
20kDa
15kDa
10kDa
KRAS
45kDa
35kDa
25kDa
β-Actin
HT29 $KRAS^{G12D}$
25kDa
20kDa
15kDa
10kDa
KRAS
45kDa
35kDa
β-Actin
Caco-2 $KRAS^{WT}$
25kDa
20kDa
15kDa
10kDa
KRAS
45kDa
35kDa
β-Actin
HT29 $KRAS^{WT}$
25kDa
20kDa
15kDa
10kDa
KRAS
45kDa
35kDa
β-Actin

Fig. 2I

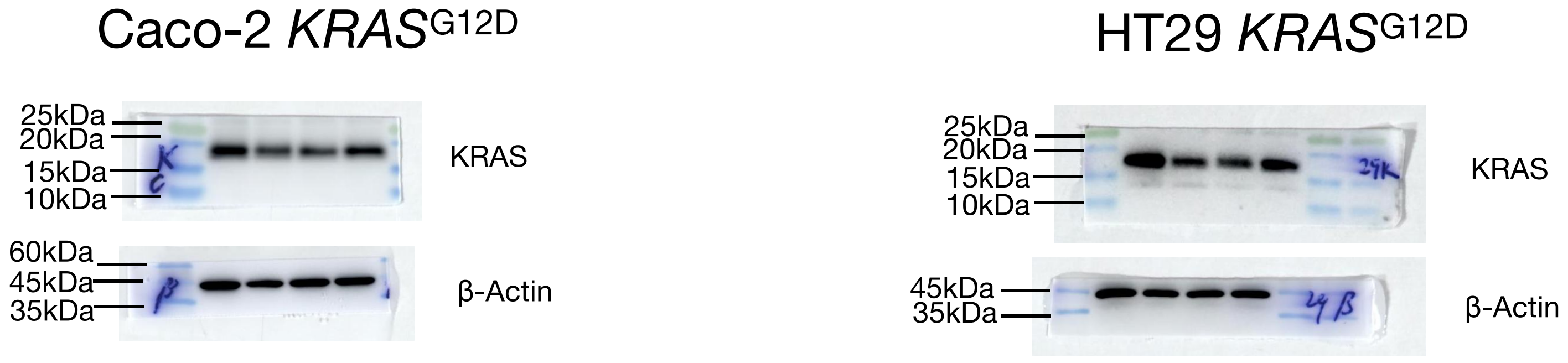
Caco-2 $KRAS^{G12D}$
25kDa
20kDa
15kDa
10kDa
KRAS
60kDa
45kDa
35kDa
β-Actin
HT29 $KRAS^{G12D}$
25kDa
20kDa
15kDa
10kDa
KRAS
45kDa
35kDa
β-Actin

Fig. 2M

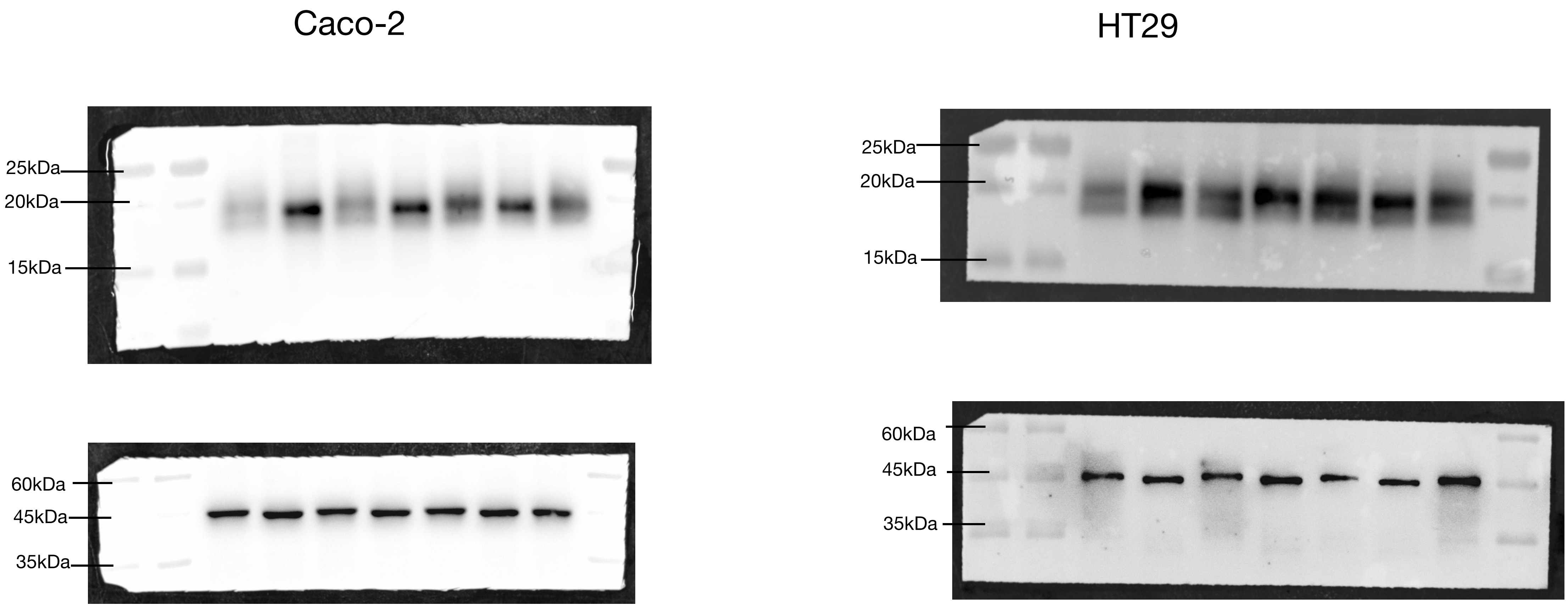
Caco-2
HT29
25kDa
20kDa
15kDa
60kDa
45kDa
35kDa
25kDa
20kDa
15kDa
60kDa
45kDa
35kDa

Fig. 3E

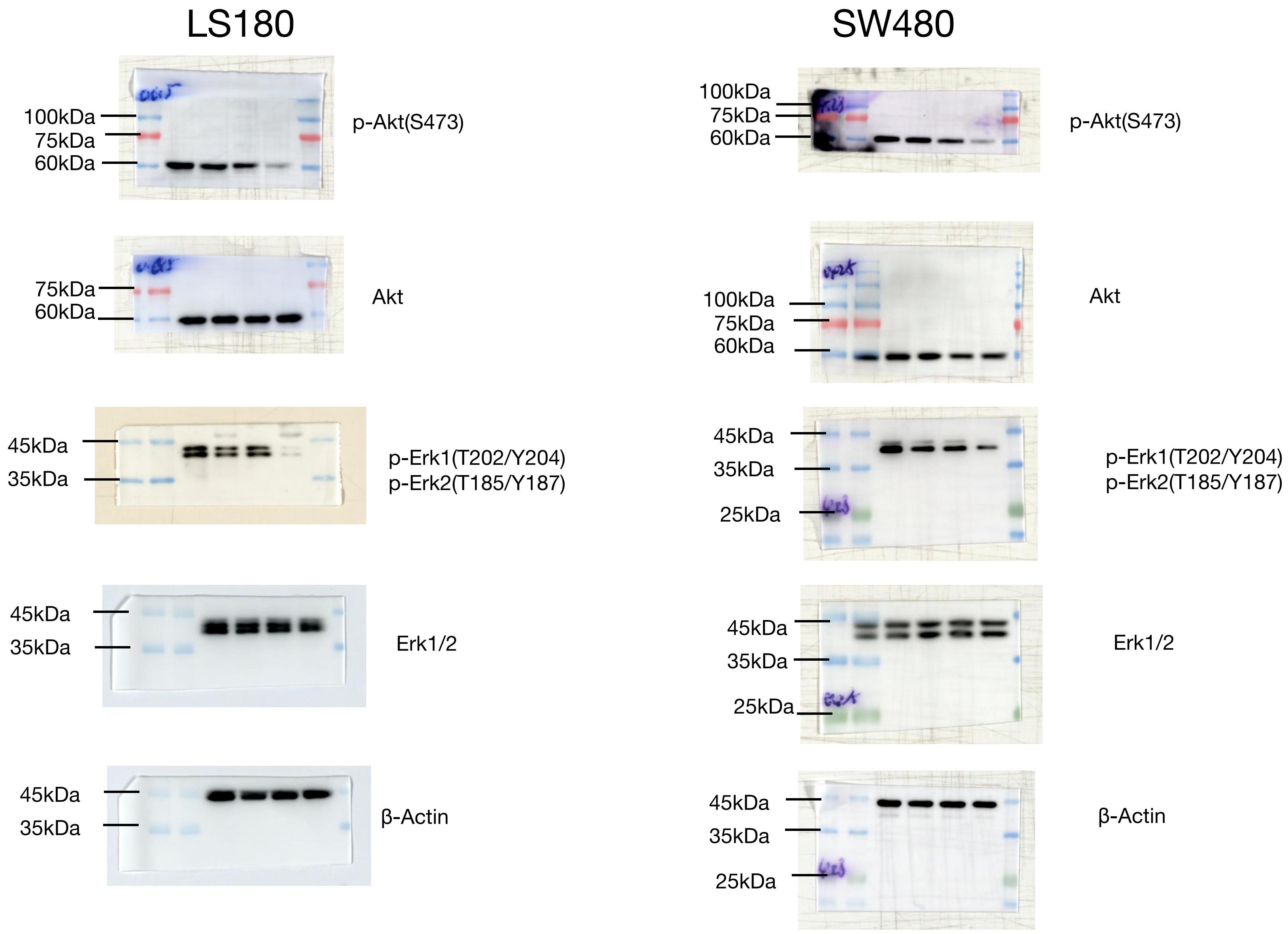

LS180
SW480
100kDa
75kDa
60kDa
p-Akt(S473)
75kDa
60kDa
Akt
45kDa
35kDa
p-Erk1(T202/Y204)
p-Erk2(T185/Y187)
45kDa
35kDa
Erk1/2
45kDa
35kDa
β-Actin
100kDa
75kDa
60kDa
p-Akt(S473)
100kDa
75kDa
60kDa
Akt
45kDa
35kDa
25kDa
p-Erk1(T202/Y204)
p-Erk2(T185/Y187)
45kDa
35kDa
25kDa
Erk1/2
45kDa
35kDa
25kDa
β-Actin

Fig. 4F

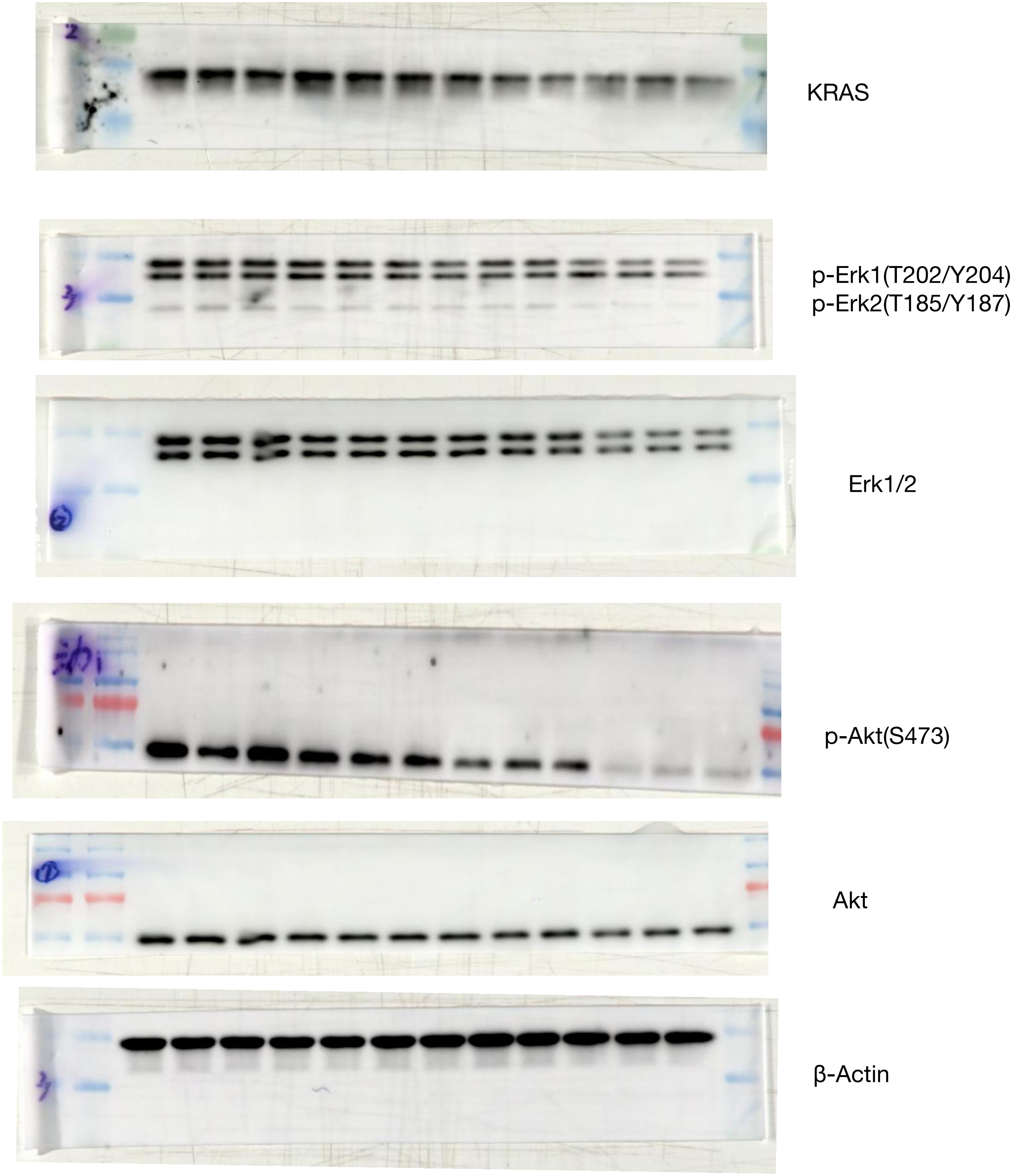
KRAS
p-Erk1(T202/Y204)
p-Erk2(T185/Y187)
Erk1/2
p-Akt(S473)
Akt
β-Actin

Fig. 6G Caco-2

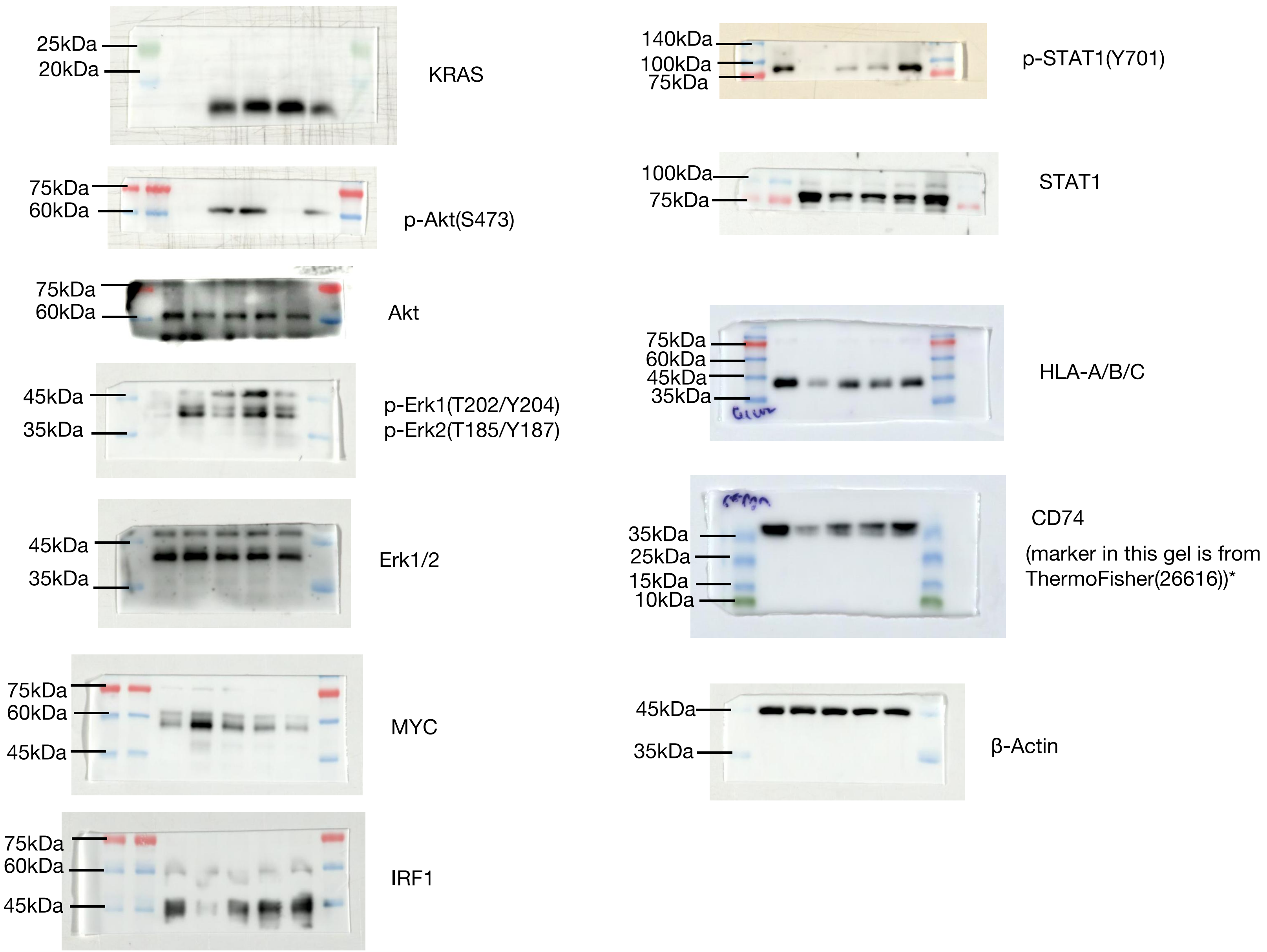

* All markers used in western blot assay were from Gene-Protein Link (P06M02), unless otherwise specified.

Fig. 6G HT29

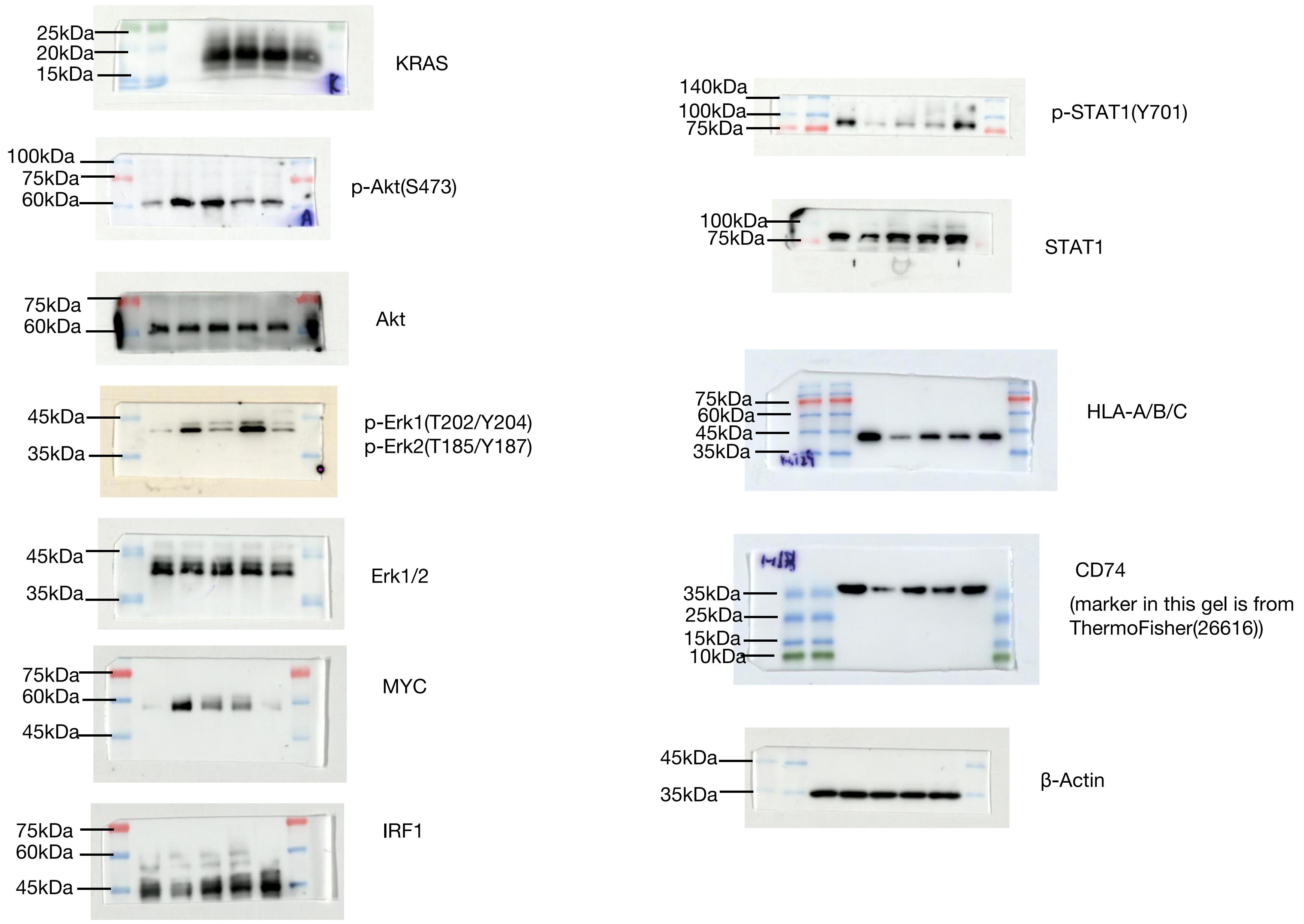
25kDa
20kDa
15kDa
KRAS
100kDa
75kDa
60kDa
p-Akt(S473)
75kDa
60kDa
Akt
45kDa
35kDa
p-Erk1(T202/Y204)
p-Erk2(T185/Y187)
45kDa
35kDa
Erk1/2
75kDa
60kDa
45kDa
MYC
75kDa
60kDa
45kDa
IRF1
140kDa
100kDa
75kDa
p-STAT1(Y701)
100kDa
75kDa
STAT1
75kDa
60kDa
45kDa
35kDa
HLA-A/B/C
35kDa
25kDa
15kDa
10kDa
CD74
(marker in this gel is from
ThermoFisher(26616))
45kDa
35kDa
β-Actin

Supplementary Fig. 1A, 1C

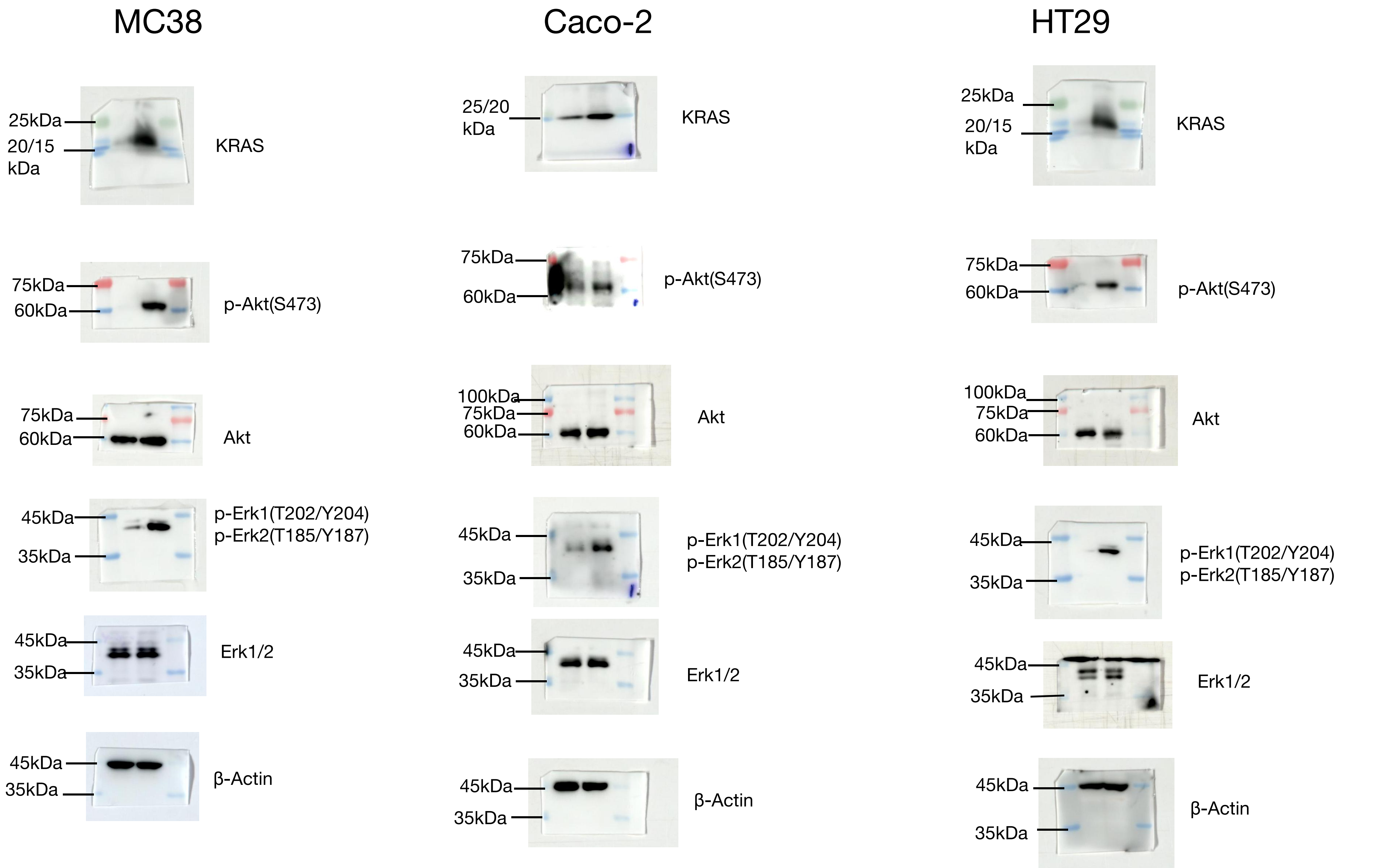

Supplementary Fig. 2H

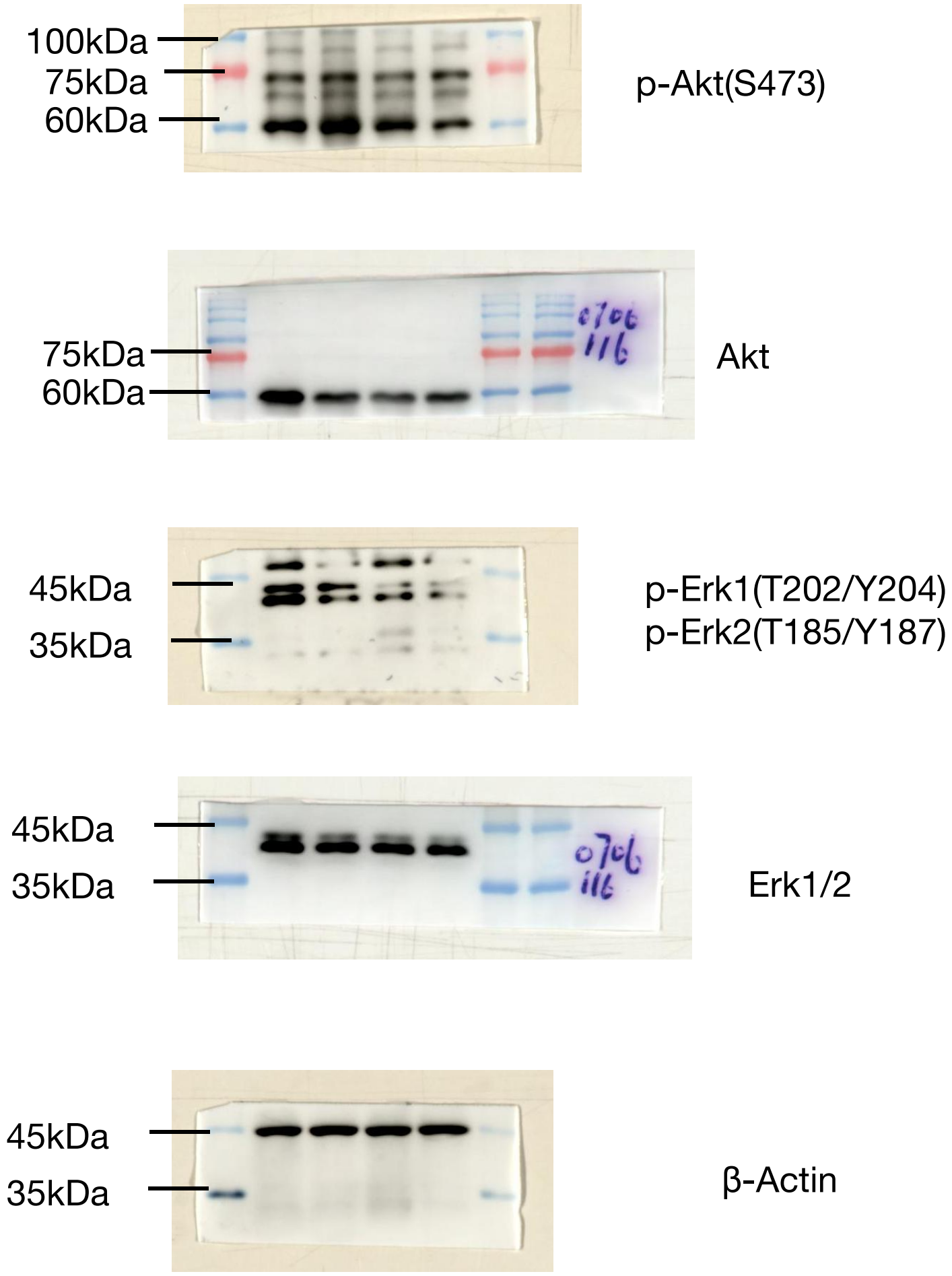

Supplementary Fig. 8E

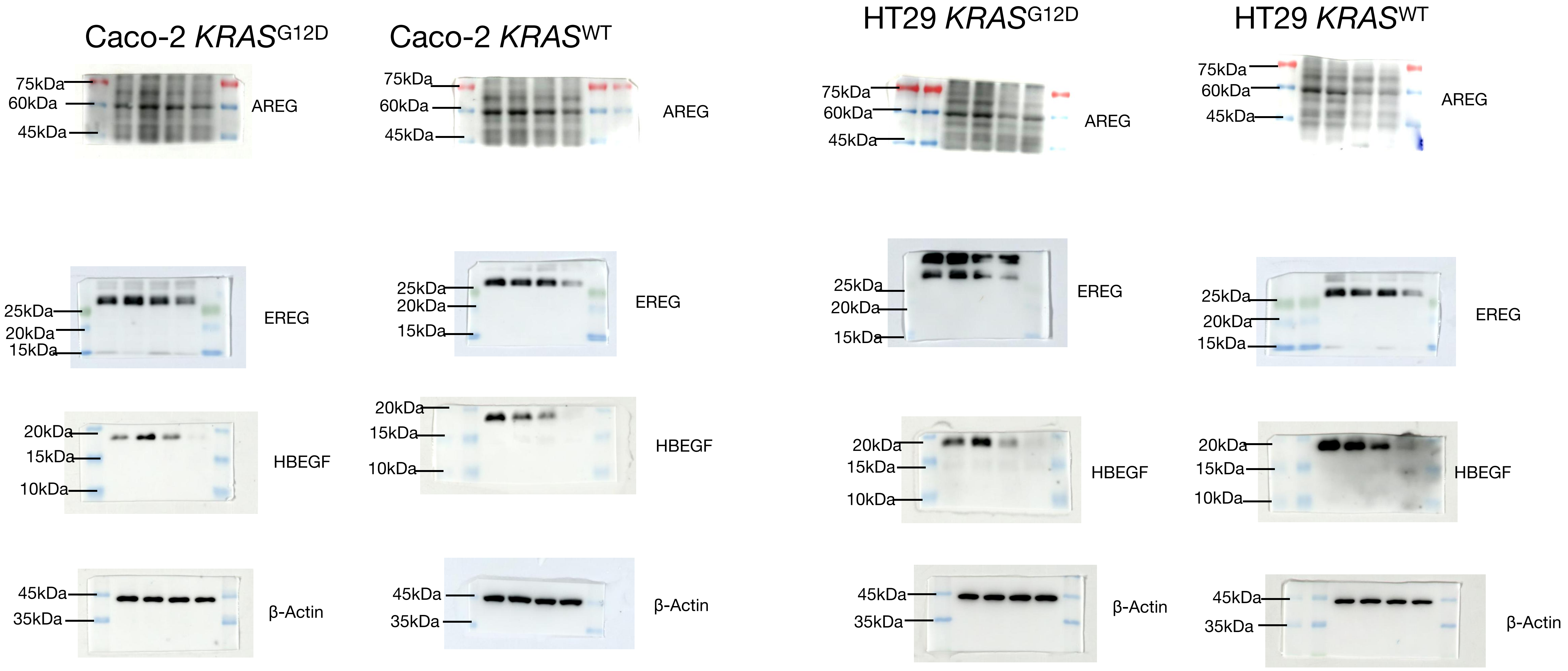

Supplement: Supplementary file 1 [file 41419_2026_9042_MOESM2_ESM.pdf]
