# Supplementary material for: Dihydroartemisinin inhibits mutant KRAS to potentiate regorafenib plus anti-PD-1 in KRAS-mutant colorectal cancer liver metastases
Source: Cell Death Dis. 2026 Jul 6;17(1):831. doi: 10.1038/s41419-026-09042-z (PMC13612556; doi:10.1038/s41419-026-09042-z)
Supplement: Supplementary file 2 [file 41419_2026_9042_MOESM1_ESM.docx]

**Supplementary Figure 1 Extended figure of Figure 1 & 2.**

(A) Validation of *Kras*^G12D^ MC38 cells construction by western blot and sanger sequencing. (B) The body weight changes of C57BL/6J mice models. (C) Validation of *KRAS*^G12D^ Caco-2 and HT29 cells construction by western blot and sanger sequencing. (D-E) DHA and Regorafenib IC50 of cell lines.

NC, Normal Control; mAb, monoclonal antibody.

**Supplementary Figure 2 Extended figure of Figure 3.**

(A-E) The synergistic analyses of regorafenib and DHA. (F) The synergistic anti-proliferation effect of regorafenib and DHA in HCT116 cells. (G) The synergistic pro-apoptosis effect of regorafenib and DHA in HCT116 cells. (H) Western blot validating the synergistic inhibition of p-Erk1/2 and p-Akt by regorafenib and DHA.

NC, Normal Control; Rego, regorafenib, DHA, dihydroartemisinin.

**Supplementary Figure 3 The therapeutic combination containing DHA has little adverse effect on mice.**

(A) The experiment workflow of BALB/c nude mice subcutaneous CRC models bearing HCT116 cells. (B) The body weight changes of BALB/c nude mice models bearing HCT116 cells. (C) The H&E staining of BALB/c nude mice liver and kidney. (D) The body weight changes of BALB/c mice CRCLM models bearing CT26 cells. (E) The H&E staining of BALB/c mice models Kidney.

NC, Normal Control; Rego, regorafenib, mAb, monoclonal antibody, DHA, dihydroartemisinin.

**Supplementary Figure 4 The cell annotation makers.**

(A) Cell annotation markers of main cell types. (B) Cell annotation markers of T cell subsets, including including Cd8^+^ effector T cells (Cd8_Te), natural killer (NK) cells, Cd4^+^ regulatory T cells (Cd4_Treg), Cd4^+^ naive T cells (Cd4_Tn) and Cd8^+^ memory T cells (Cd8_Tm). (C) Cell annotation markers of myeloid cell subsets, including including Kupffer cells, dendritic cells, Mki67^+^ macrophages (Macro_Mki67), Cxcl9^+^ macrophages (Macro_Cxcl9), Spp1^+^ macrophages (Macro_Spp1), Mrc1^+^ macrophages (Macro_Mrc1) and Ly6c^+^ monocytes (Mono_Ly6c).

**Supplementary Figure 5 Supplementary analyses of T cells. Extended figure of Figure 5.**

Feature plot showing the proliferative T cells phenotypes.

**Supplementary Figure 6 Supplementary analyses of myeloid cells. Extended figure of Figure 5.**

(A–G) GSEA of myeloid cell subsets. The representative items are displayed in **Figure 5J** and marked in red in this figure. The X-axis represents normalized enrichment score. (H) Split plot of myeloid cell in different therapeutic groups to show the CS ratio and CM ratio. (I) The cluster of neutrophils. (J–K) Feature plot of Irf1 and Cd74 expression in neutrophil. (L) GSVA of neutrophils in different therapeutic groups.

NC, Normal Control; Rego, regorafenib, mAb, monoclonal antibody, DHA, dihydroartemisinin.

**Supplementary Figure 7 The purity of CD8^+^ T cells sorted from PBLCs was detected by flow cytometry.**

The upper three panels represent the gating strategy for the isotype control, while the lower three panels correspond to the test group. From left to right, the first column displays the main cell cluster, the second column shows the single-cell cluster, and the third column illustrates the CD8⁺ cell cluster.

**Supplementary Figure 8 Regorafenib upregulates ERBB signaling ligands, AREG, EREG and HBEGF, in *KRAS*-mutant CRC.**

(A) The GSEA of tumor cells of normal control group and Rego+PD-1 mAb group based on scRNA-seq. (B) Heatmap showing *Areg*, *Ereg* and *Hbegf* expression based on scRNA-seq. (C) Heatmap showing GSVA of ERBB-associated pathways based on scRNA-seq. (D) IHC staining of AREG, EREG and HBEGF in tumor tissues of BALB/c mice models bearing CT26 cells (n=5 in each group). (E) Western blot validating the expression of AREG, EREG and HBEGF regulated by regorafenib in Caco-2 and HT29 cells. (F) IHC staining of AREG, EREG and HBEGF in tumor tissues of C57BL/6J mice models bearing MC38 cells (n=6 in each group). (G) The p-EGFR expression in *KRAS*^G12D^ and *KRAS*^WT^ cells under different treatment (n=3 replicates).

NC, Normal Control; Rego, regorafenib, mAb, monoclonal antibody, DHA, dihydroartemisinin, GSVA, Gene Set Variation Analysis, NES, Normalized Enrichment Score.

Supplementary table 1. The information of patients enrolled in bulk RNA-seq.

| ID | BRAF mutation | KRAS mutation | Microsatellite Stability | Response to regorafenib plus PD-1 inhibitor |
| --- | --- | --- | --- | --- |
| CM1 | - | - | MSS | Responder |
| CM2 | - | - | MSS |  |
| CM3 | - | - | MSS |  |
| CM4 | - | - | MSS |  |
| CM5 | - | - | MSS |  |
| CM6 | - | - | MSS |  |
| CM7 | - | - | MSS |  |
| CM8 | - | + | MSS |  |
| CM9 | - | - | MSS | Non-responder |
| CM10 | - | + | MSS |  |
| CM11 | - | - | MSS |  |
| CM12 | - | + | MSS |  |
| CM13 | - | + | MSS |  |
| CM14 | - | + | MSS |  |
| CM15 | - | + | MSS |  |
| CM16 | - | + | MSS |  |

| Targets | Manufacturers | Cat. No. | Application | Dilution |
| --- | --- | --- | --- | --- |
| KRAS | Proteintech | 12063-1-AP | WB | 1:1000 |
| p-Erk1/2 | ABclonal | AP0974 | WB | 1:1000 |
| Erk1/2 | Cell Signaling Technology | 4695T | WB | 1:1000 |
| p-Akt | Cell Signaling Technology | 9271S | WB | 1:1000 |
| Akt | Cell Signaling Technology | 9272S | WB | 1:1000 |
| MYC | Cell Signaling Technology | 13987S | WB | 1:1000 |
|  |  |  | IHC | 1:100 |
| IRF1 | ABclonal | A7692 | WB | 1:1000 |
|  |  |  | IHC | 1:500 |
| p-STAT1 | ABclonal | AP0135 | WB | 1:1000 |
|  |  |  | IHC | 1:100 |
| STAT1 | Cell Signaling Technology | 9175S | WB | 1:1000 |
| HLA-A/B/C | Proteintech | 15240-1-AP | WB | 1:5000 |
|  |  |  | IHC | 1:1000 |
| CD74 | ABclonal | A24027 | WB | 1:10000 |
|  |  |  | IHC | 1:1000 |
| AREG | Santa Cruz | SC-74501 | WB | 1:500 |
|  |  |  | IHC | 1:100 |
| EREG | Santa Cruz | SC-376284 | WB | 1:500 |
|  |  |  | IHC | 1:100 |
| HBEGF | ABclonal | A11657 | WB | 1:1000 |
|  |  |  | IHC | 1:100 |
| β-actin | ABclonal | AC026 | WB | 1:100000 |
| CD8 | ThermoFisher | 14-0081-82 | mIF（TSA）^1^ | 1:1000 |
|  |  |  | cIF | 1:1000 |
| CD68 | abcam | ab201340 | mIF（TSA） | 1:500 |
| F4/80 | abcam | ab111101 | mIF（TSA） | 1:1000 |
| CD206 | Cell Signaling Technology | 24595S | mIF（TSA） | 1:500 |
| GZMB | abcam | ab255598 | mIF（TSA） | 1:1000 |
| PRF1 | ThermoFisher | PA5-87351 | mIF（TSA） | 1:500 |
| IFNγ | Proteintech | 29788-1-AP | mIF（TSA） | 1:500 |
| CXCL9 | Proteintech | 22355-1-AP | mIF（TSA） | 1:500 |
| SPP1 | Cell Signaling Technology | 88742 | mIF（TSA） | 1:1000 |
| PanCK | Proteintech | 26411-1-AP | cIF | 1:1000 |
| Ki-67 | abcam | ab15580 | IHC | 1:100 |
| CD31 | abcam | ab182981 | IHC | 1:100 |
| p-EGFR | abcam | ab40815 | cIF | 1:1000 |

Supplementary table 2. Antibodies list

1. TSA：Tyramide signal amplification technology.

Supplementary table 3 Compounds enrolled in virtual screening

| Compound | Docking score | Ligand efficiency  (kcal/mol) |
| --- | --- | --- |
| Andrographolide | -5.408 | -0.216 |
| Allantoin | -5.402 | -0.491 |
| Dihydroartemisinin | -5.376 | -0.195 |
| Pregnenolone | -5.324 | -0.231 |
| Salicylic acid | -5.265 | -0.527 |
| Sodium Salicylate | -5.265 | -0.527 |
| Nonivamide | -5.262 | -0.251 |
| Methyl nicotinate | -5.255 | -0.525 |
| Eugenol | -5.246 | -0.437 |
| Butylphthalide | -5.21 | -0.372 |
| D-Pantothenic acid (sodium) | -5.203 | -0.347 |
| Danthron | -5.2 | -0.289 |
| D-Pantothenic acid (hemicalcium salt) | -5.183 | -0.346 |
| Flopropione | -5.159 | -0.397 |
| Vanillin | -5.128 | -0.466 |
| Diphylline | -5.12 | -0.284 |
| Niacin | -5.095 | -0.566 |
| Chlorpheniramine | -5.088 | -0.268 |
| Chlorhexidine (diacetate) | -5.087 | -0.150 |
| Resveratrol | -5.077 | -0.299 |
| DHEA | -5.076 | -0.242 |
| Pyridoxine (hydrochloride) | -5.063 | -0.422 |
| Daidzein | -5.059 | -0.266 |
| Theophylline | -5.02 | -0.386 |
| Psoralen | -4.977 | -0.355 |
| Ingenol | -4.975 | -0.199 |

| Compound | Docking score | Ligand efficiency  (kcal/mol) |
| --- | --- | --- |
| L-Ornithine | -4.955 | -0.551 |
| Trioxsalen | -4.922 | -0.290 |
| 18β-Glycyrrhetinic acid | -4.908 | -0.144 |
| Coumarin | -4.897 | -0.445 |
| L-Tryptophan | -4.883 | -0.326 |
| Benzyl alcohol | -4.834 | -0.604 |
| Metamizole (sodium) | -4.819 | -0.229 |
| Oxyphenbutazone | -4.815 | -0.201 |
| Santonin | -4.811 | -0.267 |
| Gossypol (acetic acid) | -4.806 | -0.126 |
| Methoxsalen | -4.787 | -0.299 |
| Atropine | -4.771 | -0.227 |
| Magnolol | -4.763 | -0.238 |
| Daidzin | -4.76 | -0.159 |
| Melatonin | -4.71 | -0.277 |
| Camptothecin | -4.704 | -0.181 |
| Terbutaline | -4.692 | -0.293 |
| Quinine | -4.657 | -0.194 |
| Cytisinicline | -4.621 | -0.330 |
| Thymopentin | -4.607 | -0.096 |
| Thymopentin (acetate) | -4.607 | -0.096 |
| Biotin | -4.607 | -0.288 |
| L-Threonine | -4.592 | -0.574 |
| Gossypol | -4.581 | -0.121 |
| (+)-Kavain | -4.561 | -0.268 |
| Thiamine monochloride | -4.543 | -0.252 |
| Progesterone | -4.536 | -0.197 |

| Compound | Docking score | Ligand efficiency  (kcal/mol) |
| --- | --- | --- |
| Folic acid | -4.534 | -0.142 |
| Ethylparaben | -4.524 | -0.377 |
| Adenine | -4.504 | -0.450 |
| Adenine (hydrochloride) | -4.504 | -0.450 |
| Adenine (hemisulfate) | -4.504 | -0.450 |
| Thiamine (hydrochloride) | -4.498 | -0.250 |
| Acefylline | -4.474 | -0.263 |
| Thiamine nitrate | -4.473 | -0.249 |
| Uracil | -4.439 | -0.555 |
| Malic acid | -4.436 | -0.493 |
| (-)-Securinine | -4.429 | -0.277 |
| Lactate (potassium) | -4.411 | -0.735 |
| Methyl Salicylate | -4.39 | -0.399 |
| Racanisodamine | -4.339 | -0.197 |
| L-Lactic acid | -4.255 | -0.709 |
| Monocrotaline | -4.252 | -0.185 |
| Cysteamine (hydrochloride) | -4.217 | -1.054 |
| L-Valine | -4.155 | -0.519 |
| Galanthamine | -4.154 | -0.198 |
| Galanthamine (hydrobromide) | -4.154 | -0.198 |
| L-Serine | -4.153 | -0.593 |
| L-Asparagine | -4.134 | -0.459 |
| Harringtonine | -4.072 | -0.107 |
| Hydroquinidine | -4.049 | -0.169 |
| Folinic acid | -4.026 | -0.118 |
| Erythromycin | -3.998 | -0.078 |
| Quinidine hydrochloride monohydrate | -3.989 | -0.166 |

| Compound | Docking score | Ligand efficiency  (kcal/mol) |
| --- | --- | --- |
| Demecolcine | -3.983 | -0.148 |
| Choline (bitartrate) | -3.97 | -0.567 |
| Choline (chloride) | -3.97 | -0.567 |
| L-Histidine | -3.964 | -0.360 |
| Cholic acid | -3.947 | -0.136 |
| Cholic acid (sodium) | -3.947 | -0.136 |
| Glycolic acid | -3.921 | -0.784 |
| Colchicine | -3.921 | -0.135 |
| L-Cysteine | -3.914 | -0.559 |
| L-Lysine (acetate) | -3.899 | -0.390 |
| Dehydroandrographolide | -3.898 | -0.162 |
| Eicosapentaenoic Acid | -3.862 | -0.176 |
| L-Lysine hydrate | -3.86 | -0.386 |
| L-Lysine | -3.86 | -0.386 |
| L-Lysine (hydrochloride) | -3.86 | -0.386 |
| L-Glutamic acid | -3.775 | -0.378 |
| L-Glutamic acid (monosodium salt) | -3.775 | -0.378 |
| Novobiocin | -3.758 | -0.085 |
| Guaiazulene | -3.736 | -0.249 |
| 1,3-Dihydroxyacetone | -3.722 | -0.620 |
| Berberine (chloride hydrate) | -3.713 | -0.149 |
| Berberine (chloride) | -3.713 | -0.149 |
| Berberine (hemisulfate) | -3.713 | -0.149 |
| Berberine (sulfate) | -3.713 | -0.149 |
| Deoxycholic acid | -3.676 | -0.131 |
| Deoxycholic acid sodium salt | -3.676 | -0.131 |
| Atropine (sulfate monohydrate) | -3.64 | -0.173 |

| Compound | Docking score | Ligand efficiency  (kcal/mol) |
| --- | --- | --- |
| Homoharringtonine | -3.639 | -0.093 |
| Vincamine | -3.582 | -0.138 |
| Sanguinarine (chloride) | -3.581 | -0.143 |
| Sodium citrate (dihydrate) | -3.541 | -0.272 |
| Citric acid | -3.541 | -0.272 |
| (-)-Menthol | -3.535 | -0.321 |
| Nalidixic acid | -3.533 | -0.208 |
| Diacerein | -3.517 | -0.130 |
| Ethyl linoleate | -3.513 | -0.160 |
| Thymol | -3.51 | -0.319 |
| Isocorydine | -3.494 | -0.140 |
| Citric acid (tripotassium hydrate) | -3.491 | -0.269 |
| Rotundine | -3.478 | -0.134 |
| γ-Aminobutyric acid | -3.455 | -0.494 |
| Monomethyl fumarate | -3.448 | -0.383 |
| Hyodeoxycholic acid | -3.439 | -0.123 |
| Protoporphyrin IX | -3.433 | -0.082 |
| Terpin (hydrate) | -3.428 | -0.286 |
| Oxymatrine | -3.42 | -0.180 |
| Sodium 2-oxopropanoate | -3.405 | -0.568 |
| Pyruvic acid | -3.405 | -0.568 |
| Nalidixic acid (sodium salt) | -3.401 | -0.200 |
| Tetrahydropalmatine | -3.391 | -0.130 |
| Tetrahydropalmatine (hydrochloride) | -3.391 | -0.130 |
| Indirubin | -3.364 | -0.168 |
| Novobiocin (sodium) | -3.333 | -0.076 |
| alpha-Asarone | -3.332 | -0.222 |

| Compound | Docking score | Ligand efficiency  (kcal/mol) |
| --- | --- | --- |
| L-Glutamine | -3.319 | -0.332 |
| DL-Menthol | -3.319 | -0.302 |
| Benzoic acid | -3.317 | -0.369 |
| Lidocaine (hydrochloride hydrate) | -3.313 | -0.195 |
| Vinburnine | -3.286 | -0.149 |
| Taurodeoxycholic acid | -3.279 | -0.096 |
| Tolazoline | -3.244 | -0.270 |
| Acetylcysteine | -3.204 | -0.320 |
| Menadione | -3.198 | -0.246 |
| Artemisinin | -3.196 | -0.160 |
| Taurine | -3.178 | -0.454 |
| Urea | -3.174 | -0.793 |
| Artesunate | -3.17 | -0.117 |
| Sinomenine | -3.165 | -0.132 |
| Sinomenine hydrochloride | -3.165 | -0.132 |
| Palmatine (chloride) | -3.157 | -0.121 |
| Matrine | -3.154 | -0.175 |
| Docetaxel | -3.114 | -0.054 |
| DL-alpha-Tocopherol | -3.107 | -0.100 |
| L-Phenylalanine | -3.103 | -0.259 |
| DL-Arginine | -3.074 | -0.256 |
| L-Arginine | -3.074 | -0.256 |
| L-Arginine (hydrochloride) | -3.074 | -0.256 |
| L-Arginine (L-glutamate) | -3.074 | -0.256 |
| Oleic acid | -3.055 | -0.153 |
| Sodium oleate | -3.055 | -0.153 |
| Sophoridine | -3.047 | -0.169 |

| Compound | Docking score | Ligand efficiency  (kcal/mol) |
| --- | --- | --- |
| Gamma-Linolenic acid | -3.002 | -0.150 |
| Aristolochic acid A | -2.982 | -0.119 |
| Ajmaline | -2.97 | -0.124 |
| α-Lipoic Acid | -2.956 | -0.246 |
| 2-Mercaptobenzothiazole | -2.941 | -0.294 |
| L-Carnitine | -2.924 | -0.266 |
| Vitamin K1 | -2.878 | -0.087 |
| Medroxyprogesterone acetate | -2.862 | -0.102 |
| Vinpocetine | -2.842 | -0.109 |
| (-)-Limonene | -2.825 | -0.282 |
| L-Proline | -2.767 | -0.346 |
| Ligustrazine | -2.724 | -0.272 |
| Ligustrazine (hydrochloride) | -2.724 | -0.272 |
| Artemether | -2.717 | -0.129 |
| α-Vitamin E | -2.701 | -0.087 |
| Artemotil | -2.684 | -0.122 |
| Creatine (monohydrate) | -2.677 | -0.297 |
| Topotecan (hydrochloride) | -2.666 | -0.086 |
| Podofilox | -2.65 | -0.088 |
| Chenodeoxycholic Acid | -2.642 | -0.094 |
| β-Elemene | -2.606 | -0.174 |
| Phillyrin | -2.595 | -0.068 |
| Succinic acid | -2.585 | -0.323 |
| Disodium succinate | -2.585 | -0.323 |
| Triptonide | -2.57 | -0.099 |
| Creatine | -2.556 | -0.284 |
| L-Leucine | -2.508 | -0.279 |

| Compound | Docking score | Ligand efficiency  (kcal/mol) |
| --- | --- | --- |
| Paclitaxel | -2.478 | -0.040 |
| L-Aspartic acid | -2.456 | -0.273 |
| (+)-Sparteine | -2.453 | -0.144 |
| Pregnenolone monosulfate (sodium) | -2.364 | -0.088 |
| Ajmalicine | -2.335 | -0.090 |
| (-)-Huperzine A | -2.239 | -0.124 |
| Noscapine | -2.231 | -0.074 |
| L-Isoleucine | -2.229 | -0.248 |
| Azelaic acid | -2.151 | -0.165 |
| Cabazitaxel | -2.101 | -0.035 |
| DL-Methionine | -2.086 | -0.232 |
| L-Methionine | -2.086 | -0.232 |
| Berbamine | -2.054 | -0.046 |
| Berbamine (dihydrochloride) | -2.054 | -0.046 |
| Taurodeoxycholic acid (sodium hydrate) | -2.03 | -0.060 |
| Retinoic acid | -2.007 | -0.091 |
| Fumaric acid | -1.946 | -0.243 |
| Stearic acid | -1.882 | -0.094 |
| Squalene | -1.859 | -0.062 |
| Oleanolic acid | -1.776 | -0.054 |
| L-Alanine | -1.683 | -0.280 |
| Lauric acid | -1.68 | -0.120 |
| Panaxatriol | -1.604 | -0.047 |
| Cepharanthine | -1.528 | -0.034 |
| Physostigmine (salicylate) | -1.445 | -0.072 |
| Glycine | -1.437 | -0.287 |
| (+)-Camphor | -1.354 | -0.123 |

| Compound | Docking score | Ligand efficiency  (kcal/mol) |
| --- | --- | --- |
| Camphor | -1.354 | -0.123 |
| Benzyl benzoate | -1.194 | -0.075 |
| 10-Undecenoic acid | -1.19 | -0.092 |
| Dehydroandrographolide succinate | -0.987 | -0.026 |
| 1-Hexadecanol | -0.772 | -0.045 |
| Silymarin | -0.417 | -0.417 |
| Inulin | -0.417 | -0.417 |
| Casanthranol | -0.417 | -0.417 |
| Vitamin K | -0.417 | -0.417 |
| Triacetin | -0.301 | -0.020 |

Supplementary table 4. The immune infiltration of BALB/c mice models assessed by Cibersort.

| Cell type | Rego+PD-1 mAb (%) | Rego+PD-1 mAb+DHA  (%) |
| --- | --- | --- |
| B cell naive | 13.7832703 | 8.4007996 |
| B cell memory | 0 | 0 |
| B cell plasma | 10.0964461 | 2.3738699 |
| T cell CD8^+^ | 3.9832762 | 8.2882043 |
| T cell CD4^+^ naive | 1.6186024 | 0 |
| T cell CD4^+^ memory resting | 0 | 3.3215246 |
| T cell CD4^+^ memory activated | 0 | 0 |
| T cell follicular helper | 0.9938115 | 1.6190696 |
| T cell regulatory (Tregs) | 1.2158036 | 1.8822104 |
| T cell gamma delta | 0 | 0 |
| NK cell resting | 5.9113297 | 7.0048071 |
| NK cell activated | 0 | 0.3340462 |
| Monocyte | 0 | 1.4079508 |
| Macrophage M0 | 2.7130965 | 6.1267854 |
| Macrophage M1 | 4.9545453 | 9.0722167 |
| Macrophage M2 | 8.3117113 | 0 |
| Myeloid dendritic cell resting | 33.3190021 | 41.5032204 |
| Myeloid dendritic cell activated | 0 | 0 |
| Mast cell activated | 0 | 0 |
| Mast cell resting | 5.3785519 | 0 |
| Eosinophil | 0 | 0 |
| Neutrophil | 7.720553 | 8.6652951 |
